# C-reactive protein and cancer risk: a pan-cancer study of prospective cohort and Mendelian randomization analysis

**DOI:** 10.1186/s12916-022-02506-x

**Published:** 2022-09-19

**Authors:** Meng Zhu, Zhimin Ma, Xu Zhang, Dong Hang, Rong Yin, Jifeng Feng, Lin Xu, Hongbing Shen

**Affiliations:** 1 Department of Thoracic Surgery, Jiangsu Cancer Hospital & Jiangsu Institute of Cancer Research & The Affiliated Cancer Hospital of Nanjing Medical University, Baiziting 42, Nanjing, China; 2 Department of Epidemiology, Center for Global Health, School of Public Health, Nanjing Medical University, 101 Longmian Rd, Nanjing, 211166 China; 3 Department of Epidemiology, School of Public Health, Southeast University, Nanjing, China; 4 Department of Medical Oncology, Jiangsu Cancer Hospital &Jiangsu Institute of Cancer Research & The Affiliated Cancer Hospital of Nanjing Medical University, Baiziting 42, Nanjing, China

**Keywords:** C-reactive protein, Cancer risk, Cohort study, Mendelian randomization analysis, Non-linear Mendelian randomization

## Abstract

**Background:**

Although observational studies have reported associations between serum C-reactive protein (CRP) concentration and risks of lung, breast, and colorectal cancer, inconsistent or absent evidences were showed for other cancers. We conducted a pan-cancer analysis to comprehensively assess the role of CRP, including linearity and non-linearity associations.

**Methods:**

We analyzed 420,964 cancer-free participants from UK Biobank cohort. Multivariable-adjusted Cox proportional hazards model was conducted to evaluate the observed correlation of CRP with overall cancer and 21 site-specific cancer risks. Furthermore, we performed linear and non-linear Mendelian randomization analyses to explore the potential causal relation between them.

**Results:**

During a median follow-up period of 7.1 years (interquartile range: 6.3, 7.7), 34,979 incident cancer cases were observed. Observational analyses showed higher CRP concentration was associated with increased risk of overall cancer (hazard ratio (HR) = 1.02, 95% CI: 1.01, 1.02 per 1mg/L increase, *P* < 0.001). There was a non-linear association between CRP and overall cancer risk with inflection point at 3mg/L (false-discovery rate adjust (FDR-adjusted) *P*_overall_ < 0.001 and FDR-adjusted *P*_non-linear_ < 0.001). For site-specific cancer, we observed positive linear associations for cancers of esophagus and stomach (FDR-adjusted *P*_overall_ < 0.050 and FDR-adjusted *P*_non-linear_ > 0.050). In addition, we also observed three different patterns of non-linear associations, including “fast-to-low increase” (head and neck, colorectal, liver, lung, kidney cancer, and non-Hodgkin lymphoma), “increase-to-decrease” (breast cancer), and “decrease-to-platform” (chronic lymphocytic leukemia). Furthermore, the inflection points of non-linear association patterns were consistently at around 3mg/L. By contrast, there was no evidence for linear or non-linear associations between genetically predicted CRP and risks of overall cancer or site-specific cancers.

**Conclusions:**

Our results indicated that CRP was a potential biomarker to assess risks of overall cancer and 12 site-specific cancers, while no association were observed for genetically-predicted CRP and cancer risks.

**Supplementary Information:**

The online version contains supplementary material available at 10.1186/s12916-022-02506-x.

## Background

Inflammation has been demonstrated as the seventh hallmark of cancer [1]. It has been suggested that the state of chronic low-grade inflammation predisposed a person to cancer by building up an inflammatory microenvironment [2]. Although C-reactive protein (CRP) is a classical acute phase reactant protein from pentraxin family, a moderate increase of CRP level had been observed in chronic inflammatory states [3]. Previous studies have reported associations between serum CRP concentration and cancers of lung [4, 5], breast [6], and colorectal [7], while showing inconsistent or absent evidences for the associations of other cancer. This indicated the necessity of a pan-cancer analysis to systematically evaluate the associations of CRP and cancer risk, especially in prospective studies. In addition, the causal relationship between CRP and cancer risk remained to be explored due to the potential unmeasured confounders or reverse causality in observational studies.

Mendelian randomization (MR) analysis has been widely used to explore the causal associations between risk factors and diseases [8, 9]. Previous MR analysis has showed that the genetically elevated CRP concentration was probably to be a causal factor for gallbladder cancer [10], while not for cancers of colorectal [8], breast [11], or prostate [12]. The heritability of CRP was estimated to range between 25 and 40% [13]. However, only a small number of single nucleotide polymorphisms (SNPs) have been identified to construct the genetic instrument of CRP in previous studies [14, 15]. Recently, additional CRP-related susceptibility loci have been identified with the accumulation of sample size, which will help construct a more effective genetic instrumental variable [16].

Traditional MR analysis tested the hypothesis of linearity between exposure and outcome, while ignoring the possible non-linear relationship [17]. Recently, a new method was proposed to assess the potential non-linear J- or U-shape effects in MR analysis [17], which has been successfully used in the investigation of diastolic blood pressure and the risk of myocardial infarction [18]. The non-linear MR analysis took the strategy of conditioning on quantiles of instrumental variable and generated localized average causal effect (LACE) estimates. Hence, exploring the non-linear relationship between CRP concentration and cancer risk in observational studies and MR analysis, simultaneously, might provide new knowledge for understanding CRP concentration and cancer risk.

In this study, based on a large-scale prospective cohort study-the UK Biobank, we performed a pan-cancer analysis to assess the linear and non-linear associations between CRP and cancer risk. Furthermore, with the traditional MR and a state-of-the-art non-linear MR, we explored the linear and non-linear relationships between genetically predicted CRP and cancer risk simultaneously.

## Methods

### Study population

All data included in this analysis were obtained from UK Biobank. The UK Biobank is a large-scale cohort, including 502,507 participants recruited from 22 assessment centers throughout the UK during 2006–2010 [19]. All participants completed a written informed consent form, a self-completed touch-screen questionnaire, a brief computer-assisted interview, and physical measures. Meanwhile, biological samples including blood were collected through strict quality control during the baseline period from different centers [19].

In this study, we excluded 46,533 patients with cancer at baseline, 30,035 participants without CRP information, and 4975 individuals without genetic data. Finally, a total of 420,964 participants were included in this study.

### Assessment of exposure, outcome, and covariates

The CRP concentration was measured by immunoturbidimetric-high sensitivity analysis on Beckman Coulter AU5800 at baseline, with a range from 0.08 to 79.96 mg/L. The outliers were capped by the 1st percentile (Q1) or 99th percentile (Q99) of CRP level. A special detail of collection and processing of blood sample has been described elsewhere [20].

Cancer outcomes were defined based on the ICD10 coding and obtained from the national cancer registry. The follow-up time referred to the period from baseline enrollment to the first diagnosis of cancer, the first registration of cancer or loss, or end of follow-up (31 October 2015 for Scotland and 31 March 2016 for England and Wales). After excluding site-specific cancer with less than 100 incident cases, we finally included overall cancer and 21 site-specific cancers in this study (Additional file 2: Table S1).

Variables that might affect the association between CRP and cancer risk based on previous studies were considered as covariates in our analysis, including age, family cancer history, body mass index (BMI), height, smoking status, alcohol use, and physical activity for both male and female, as well as menopausal, oral contraceptive use, and hormone replacement therapy for female [21]. Besides, we also included sex, ethnic, education, Townsend deprivation index, and assessment center as covariates. These covariates were collected using a touchscreen questionnaire or measured by trained staffs at baseline, and no covariates had more than 2.0% of missing values (Additional file 2: Table S2). The missing values on continued covariates were replaced with the sex-specific mean value of each variable. And missing values on categorical covariates were considered as “unknown” category.

### Genotyping

Genome-wide genotyping was performed using the Affymetrix UK BiLEVE Axiom array or the Affymetrix UK Biobank Axiom array. The two arrays share 95% of the markers. Imputation was performed with SHAPEIT3 and IMPUTE3 based on merged UK10K and 1000 Genomes phase3 panels [22]. Markers with minor allele frequency > 0.001 and Info score > 0.3 were retained in UK Biobank. Detail information on genotype quality, quality control, and genotype imputation has been described in previous study [22].

### Genetic instrument for serum CRP level

A total of 52 susceptibility loci associated with serum CRP concentration have been identified in a previous GWAS [16], which was used to construct the genetic instrument of CRP by calculating the weighted genetic risk score (wGRS). The genetic instrument was strongly associated with serum CRP concentration with an *F* statistic of 216 and could explain 2.6% of the variance of CRP in this study (Additional file 1). In addition, five SNPs associated with both colorectal cancer and serum CRP concentration were further excluded in the sensitivity analysis to evaluate the validity of the instruments (Table S3 in the Additional file 2).

### Statistical analysis

Cox proportional hazards regression was conducted to assess the association between CRP and cancer risk. Schoenfeld residuals and log-log inspection were used to test the assumption of proportional hazards. The time scale in the Cox PH regression was from the enrolment until the time of cancer diagnosis, death, withdrawal from study, or the end of follow-up, whichever came first. We estimated the hazard ratio (HR) associated with CRP (per 1 mg/L increase) for each site-specific cancer in all eligible participants and re-evaluated the HRs by dividing participants into low CRP level (≤ 3mg/L) and high CRP level (>3 mg/L) [23]. We further applied restricted cubic spline analysis to explore the possibly non-linear association shapes between serum CRP concentration and cancer risk*.* To balance the best fitting and over fitting in the splines for cancer, the number of knots were tested from three to five, and we chose that with the lowest value of Akaike information criterion (AIC); if the same AIC was observed for different knots, the lowest number of knots was chosen [24]. Except for lung cancer (4 knots at the 5th, 35th, 65th, and 95th percentile of CRP), we fitted the models of overall cancer and other site-specific cancer with 3 knots at the 10th, 50th, and 90th percentile of CRP. We used a likelihood ratio test to calculate *P*-value for non-linearity by comparing the model with only a linear term against the model with linear and cubic spline terms [25]. We further performed subgroup analyses to assess potential effect modification by age, sex, and smoking status using likelihood ratio tests. To examine the robustness of our results, we performed several sensitivity analyses: (1) re-analysis the association between log-transformed CRP level and cancer risk, (2) exclusion or only inclusion of participants diagnosed with cancer within the first two follow-up to avoid the potential reverse causality, (3) exclusion of participants with CRP level of >10 mg/L to avoid the effect of acute serious infection, (4) additionally adjusted for cardiovascular disease and diabetes, and (5) additionally adjusted for regular use of aspirin and ibuprofen.

The potential linear and non-linear causal associations between CRP concentration and cancer risk were simultaneously evaluated in this study. To evaluate the potential linear associations, we performed a two-stage MR analysis. In the first stage, we estimate the fitted values using a regression of CRP against wGRS, and in second stage, the predicted value was further fitted in a Cox regression model with cancer risk. Covariates, including age at baseline, sex, and the top 10 genetic principal components, were adjusted in both stages. In addition, several sensitivity analyses were also performed in the analysis: (1) we re-estimated the causal associations between log-transformed CRP level and cancer risks, (2) two-stage MR was only conducted in participants of British ancestry, and (3) rs2794520, the strongest SNP in previous GWAS, was used as an instrument variable to minimize the possibility of introducing horizontal pleiotropy [16].

For non-linear MR analysis, the sample was stratified into three strata according to residual CRP (the CRP minus the genetically predicted CRP). Next, we assessed the exposure-outcome associations using the piecewise linear method within each stratum, by contributing a line piece whose gradient is the LACE [17]. Two tests were then applied for non-linear hypothesis: (1) a heterogeneity test using Cochran’s Q statistic to analyze the difference between the LACE estimates and (2) a trend test, which conducted a meta-regression of the LACE estimates against the mean value of the CRP in each stratum.

All analyses were performed with R (version 3.6.0), and the two-sided *P* value of <0.05 was considered as statistically significant. To avoid the inflation of false-positive findings, we calculated the false-discovery rate (FDR) adjusted *P* values across the main analyses. Linear and non-linear MR analyses were conducted using the “MendelianRandomization” and “nlmr” packages, respectively.

## Results

### Baseline characteristics

The baseline characteristics of participants are illustrated in Table 1. Out of 420,964 eligible participants, 34,979 incident cancer events were diagnosed during a median follow-up of 7.1 years (interquartile range, IQR: 6.3–7.7). Participants with incident cancer had higher CRP concentration (2.7 ± 3.6 mg/L) than those without incident cancer (2.4 ± 3.3 mg/L).

**Table 1:** Baseline characteristics of study participants by incident overall cancer

	Overall (***N***=420,964)	No cancer (***N***=385,985)	Incident cancer (***N***=34,979)
**CRP at baseline,(mg/L)**	2.4 ± 3.3	2.4 ± 3.3	2.7 ± 3.6
**Age at baseline,(years)**	56.2 ± 8.1	55.9 ± 8.1	60.2 ± 6.8
**Male,*n*(%)**	197,223 (46.9)	178,749 (46.3)	18,474 (52.8)
**Ethnic,*n*(%)**			
White	395,650 (94.0)	361,760 (93.7)	33,890 (96.9)
Asian	9994 (2.4)	9632 (2.5)	362 (1.0)
African	6829 (1.6)	6557 (1.7)	272 (0.8)
Mixed background	2551 (0.6)	2427 (0.6)	124 (0.4)
Unknown	5940 (1.4)	5609 (1.5)	331 (0.9)
**Education,*n*(%)**			
No degree	278,328 (66.1)	254,225 (65.9)	24,103 (68.9)
Degree	137,610 (32.7)	127,166 (33.0)	10,444 (29.9)
Unknown	5026 (1.2)	4594 (1.1)	432 (1.2)
**Townsend deprivation index**	−1.3 ± 3.1	−1.3 ± 3.1	−1.5 ± 3.0
**Standing height, (cm)**	168.6 ± 9.3	168.6 ± 9.3	169.3 ± 9.1
**BMI, (kg/m**^**2**^**)**	27.4 ± 4.8	27.4 ± 4.8	27.6 ± 4.7
**Smoking status,*n*(%)**			
Never	230,940 (54.9)	213,858 (55.4)	17,082 (48.8)
Previous	143,521 (34.1)	129,772 (33.6)	13,749 (39.3)
Current	44,385 (10.5)	40,431 (10.5)	3954 (11.3)
Unknown	2118 (0.5)	1924 (0.5)	194 (0.6)
**Alcohol use,*n*(%)**			
Never	18,583 (4.4)	17,311 (4.5)	1272 (3.6)
Previous	14,892 (3.5)	13,547 (3.5)	1345 (3.9)
Current	386,411 (91.8)	354,112 (91.7)	32,299 (92.3)
Unknown	1078 (0.3)	1015 (0.3)	63 (0.2)
**Physical activity (MET/week),*n*(%)**			
<600	63,447 (15.0)	58,199 (15.1)	5248 (15.0)
600–3000	252,403 (60.0)	231,494 (60.0)	20,909 (59.8)
≥3000	105,114 (25.0)	96,292 (24.9)	8822 (25.2)
**Having family cancer history,*n*(%)**	145,198 (34.5)	131,509 (34.1)	13,689 (39.1)
**Menopausal,*n*(%)**^a^			
No	55,655 (24.9)	53,240 (25.7)	2,415 (14.6)
Yes	133,190 (59.5)	121,674 (58.7)	11,516 (69.8)
Not sure	34,209 (15.3)	31,665 (15.3)	2544 (15.4)
Unknown	687 (0.3)	657 (0.3)	30 (0.2)
**Oral contraceptive use,*n*(%)**^a^			
Never	41,599 (18.6)	38,025 (18.4)	3574 (21.7)
Ever	181,103 (80.9)	168,223 (81.2)	12,880 (78.0)
Unknown	1039 (0.5)	988 (0.5)	51 (0.3)
**Hormone replacement therapy,*n*(%)**^a^			
Never	138,909 (62.1)	130,148 (62.8)	8761 (53.1)
Ever	83,657 (37.4)	75,983 (36.7)	7674 (46.5)
Unknown	1175 (0.5)	1105 (0.5)	70 (0.4)
**Assessment center,*n*(%)**			
East Midlands	28,471 (6.8)	26,106 (6.8)	2365 (6.8)
London	58,162 (13.8)	54,306 (14.1)	3856 (11.0)
North Eastern England	49,043 (11.7)	44,883 (11.6)	4160 (11.9)
North West England	88,420 (21.0)	80,906 (21.0)	7514 (21.5)
Scotland	55,745 (13.2)	50,767 (13.2)	4978 (14.2)
South East England	12,216 (2.9)	10,874 (2.8)	1342 (3.8)
South west England	36,372 (8.6)	33,252 (8.6)	3120 (8.9)
Wales	17,998 (4.3)	16,483 (4.3)	1515 (4.3)
West Midlands England	37,451 (8.9)	34,445 (8.9)	3006 (8.6)
Yorkshire and the Humber	37,086 (8.8)	33,963 (8.8)	3123 (8.9)

^a^Analysis was only conducted among women

Data are presented as mean ± standard deviation for continuous variables and *n* (%) for categorical variables

### Observational association evaluation

The increase of serum CRP concentration was associated with incident events of overall cancer (HR = 1.02, 95% CI: 1.01–1.02 per 1mg/L increase, *P* < 0.001). Consistently, similar associations were observed for cancers of head and neck, esophagus, stomach, colorectal, liver, lung, uterus, kidney, non-Hodgkin lymphoma, and multiple myeloma (Additional file 2: Table S4).

**Fig. 1:**
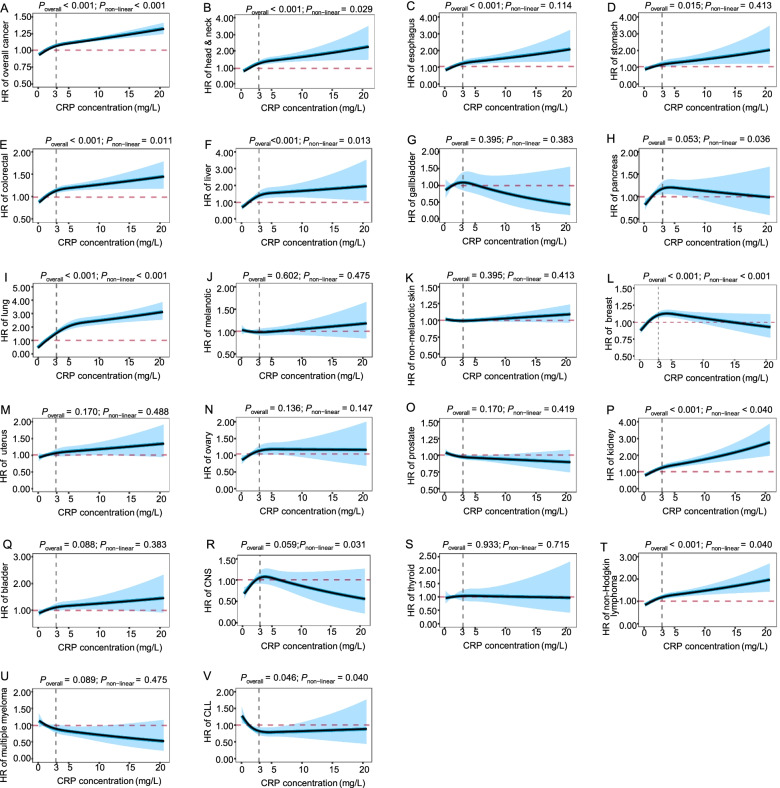
Analysis of the shape of the relationship between CRP and cancer outcomes using restricted cubic spline based on observational data. All *P* values in the figure are false-discovery rate adjusted *P* values. Adjusted for age, sex (female, male), ethnic (White, Asian, African, mixed background, unknown), education (no degree, degree, unknown), Townsend deprivation index, standing height, BMI, smoking status (never, previous, current, unknown), alcohol use (never, previous, current, unknown), physical activity (<600 MET/week, 600–3000 MET/week, ≥3000 MET/week), family cancer (no, yes), and assessment center. Additionally, adjusted for menopausal (no, yes, not sure, unknown), oral contraceptive use (never, ever, unknown), and hormone replacement therapy (never, ever, unknown) for female. CNS, central nervous system; CLL, chronic lymphocytic leukemia

To explore potential non-linear associations between CRP concentration and cancer risk, we estimated the associations with restricted cubic spline analysis. As shown in Fig. 1, a non-linear association was also observed between CRP concentration and overall cancer risk with inflection point at 3mg/L (FDR-adjusted *P*_overall_ < 0.001, and FDR-adjusted *P*_non-linear_ < 0.001). For site-specific cancer, we observed three different patterns of non-linear associations along with the increase of CRP concentration (FDR-adjusted *P*_overall_ < 0.05, and FDR-adjusted *P*_non-linear_ < 0.05), including (1) “fast-to-low increase” of risks for cancers of head and neck, colorectal, liver, lung, kidney cancer, and non-Hodgkin lymphoma; (2) “increase-to-decrease” of risk for breast cancer; and (3) “decrease-to-platform” of risk for chronic lymphocytic leukemia (CLL). In addition, we also observed positive linear associations for cancers of esophagus and stomach along with the increase of CRP concentration (FDR-adjusted *P*_overall_ < 0.050, and FDR-adjusted *P*_non-linear_ > 0.050). In sensitivity analyses, we observed a consistent inflection point of 3mg/L by exclusion of incident cancer cases diagnosed within the first 2 years of follow-up (Additional file 3: Figure S1). Furthermore, we also observed a non-linear association with inflection point at 1mg/L after log-transformed of CRP levels (Additional file 3: Figure S2).

Even though different non-linear association patterns were observed for site-specific cancer, the inflection points of the associations were consistently at around 3mg/L, which was in consistent with the risk stratification of cardiovascular disease by the American Heart Association [24]. Therefore, we further re-estimated the associations in participants of low CRP level (≤ 3mg/L) and high CRP level (>3 mg/L), respectively. Univariable analysis shows significant heterogeneities along with the increase of CRP between the two subgroups (Additional file 2: Table S5). After adjusting for covariates, statistically significant heterogeneities (FDR-adjusted *P*_heterogeneity_ < 0.05) of the association effects were also observed for overall cancer, colorectal, lung, and breast cancer (Table 2). Sensitivity analyses indicated that the associations were robust by re-evaluating the association between log-transformed CRP level and cancer risk (Additional file 2: Table S6), additionally adjusting for cardiovascular and diabetes diseases (Additional file 2: Table S7), additionally adjusting for the regular use of drugs that may affect the CRP concentration (Additional file 2: Table S8), excluding or only including individuals with incident cancer within the first two of follow-up (Additional file 2: Table S9 and S10), and excluding individuals with CRP > 10 mg/L (Additional file 2: Table S11).

**Table 2:** Hazard ratios (HRs) for cancer outcomes per 1 mg/L higher CRP concentration based on observation data

	Low CRP (≤ 3mg/L)					High CRP (>3 mg/L)				FDR-adjusted ***P***_***heterogeneity***_
	No (incident cases)	Person years	HR (95%CI)	***P***		No (incident cases)	Person years	HR (95%CI)	***P***	
**Overall cancer**	327,031 (26,173)	2,237,479	1.04 (1.03,1.06)	<0.001	<0.001	93,933 (8,806)	635,846	1.01 (1.01,1.01)	<0.001	<0.001
**Head & neck (C00–14)**	301,209 (351)	2,137,461	1.08 (0.93,1.25)	0.318	0.663	85,283 (156)	603,797	1.02 (0.99,1.05)	0.180	0.482
**Esophagus (C15)**	301,201 (344)	2,137,476	1.14 (0.99,1.32)	0.078	0.434	85,273 (146)	603,701	1.04 (1.01,1.07)	0.014	0.217
**Stomach (C16)**	301,087 (229)	2,136,975	1.05 (0.87,1.26)	0.614	0.873	85,232 (106)	603,559	1.03 (0.99,1.07)	0.154	0.833
**Colorectal (C18–20)**	303,003 (2,145)	2,144,527	1.11 (1.04,1.18)	0.001	0.033	85,917 (791)	605,899	1.02 (1.00,1.03)	0.027	0.006
**Liver (C22)**	301,037 (179)	2,136,852	1.20 (0.98,1.47)	0.080	0.235	85,230 (103)	603,594	1.01 (0.97,1.05)	0.788	0.096
**Gallbladder (C23–24)**	300,952 (94)	2,136,473	0.87 (0.65,1.17)	0.351	0.873	85,172 (45)	603,354	0.90 (0.82,0.99)	0.035	0.808
**Pancreas (C25)**	301,268 (410)	2,137,813	1.14 (0.99,1.30)	0.066	0.163	85,286 (159)	603,798	0.99 (0.95,1.02)	0.510	0.052
**Lung (C33–34)**	301,995 (1,137)	2,140,855	1.26 (1.16,1.36)	<0.001	<0.001	85,882 (755)	606,121	1.03 (1.02,1.05)	<0.001	<0.001
**Melanoma (C43)**	301,966 (1,111)	2,140,358	0.98 (0.9,1.07)	0.655	0.663	85,437 (310)	604,460	1.01 (0.99,1.04)	0.238	0.464
**Non-melanotic skin (C44)**	309,450 (8,597)	2,170,575	1.01 (0.97,1.04)	0.729	0.945	87,430 (2,305)	612,401	1.01 (1.00,1.02)	0.128	0.945
**Breast (C50)**	160,803 (3,644)	1,130,896	1.07 (1.02,1.13)	0.003	0.007	51,446 (1,371)	361,233	0.99 (0.98,1.00)	0.137	0.001
**Uterus (C54-55)**	157,651 (491)	1,119,136	1.13 (1.00,1.28)	0.050	0.226	50,382 (306)	357,326	1.01 (0.99,1.04)	0.321	0.082
**Ovary (C56)**	157,548 (388)	1,118,715	1.12 (0.97,1.29)	0.126	0.304	50,223 (147)	356,717	1.00 (0.96,1.04)	0.993	0.138
**Prostate (C61)**	148,034 (4,337)	1,035,523	0.99 (0.95,1.03)	0.649	0.824	36,092 (1,043)	250,894	1.00 (0.99,1.01)	0.811	0.712
**Kidney (C64)**	301,320 (463)	2,137,990	1.13 (0.99,1.28)	0.066	0.477	85,362 (235)	604,047	1.05 (1.03,1.07)	<0.001	0.278
**Bladder (C67)**	301,270 (412)	2,137,591	1.05 (0.91,1.20)	0.524	0.819	85,281 (154)	603,785	1.01 (0.98,1.05)	0.394	0.670
**CNS (C70–72)**	301,185 (327)	2,137,370	1.14 (0.98,1.33)	0.092	0.154	85,232 (105)	603,587	0.96 (0.92,1.01)	0.113	0.037
**Thyroid (C73)**	301,031 (173)	2,136,726	1.07 (0.86,1.32)	0.553	0.819	85,179 (52)	603,385	1.01 (0.96,1.07)	0.617	0.656
**Non-Hodgkin lymphoma (C82–85, 96)**	301,634 (776)	2,139,208	1.08 (0.98,1.20)	0.123	0.477	85,447 (320)	604,276	1.02 (1.00,1.05)	0.040	0.282
**Multiple myeloma (C90)**	301,172 (315)	2,137,379	0.89 (0.76,1.05)	0.182	0.625	85,211 (84)	603,538	0.96 (0.91,1.02)	0.178	0.398
**CLL (C91)**	301,166 (308)	2,137,248	0.83 (0.70,0.98)	0.033	0.154	85,205 (78)	603,491	1.00 (0.95,1.05)	0.924	0.042

Adjusted for age, sex (female, male), ethnic (White, Asian, African, mixed background, unknown), education (no degree, degree, unknown), Townsend deprivation index, standing height, BMI, smoking status (never, previous, current, unknown), alcohol use (never, previous, current, unknown), physical activity (<600 MET/week, 600–3000 MET/week, ≥3000 MET/week), family cancer (no, yes), and assessment center

Further adjusted for menopausal (no, yes, not sure, unknown), oral contraceptive use (never, ever, unknown), and hormone replacement therapy (never, ever, unknown) for female

*CNS* central nervous system, *CLL* chronic lymphocytic leukemia

Based on CRP concentration, participants were divided into three groups according to American Heart Association: low (< 1.0 mg/L), intermediate (1.0 to 3.0 mg/L), and high (> 3.0 mg/L). Compared with individuals at low CRP concentration, those in the intermediate and high CRP concentration had a higher risk of overall cancer, with HRs of 1.05 (95%CI: 1.02, 1.07; *P* < 0.001) and 1.15 (95%CI: 1.12, 1.18; FDR-adjusted *P* < 0.001), respectively (Table 3). Similar association results were observed for cancers of head and neck, colorectal, liver, lung, breast, ovary, kidney, and non-Hodgkin lymphoma (FDR-adjusted *P*_trend_ < 0.05). On the contrary, individuals at high CRP concentration were at decreased risks for multiple myeloma and CLL (FDR-adjusted *P*_trend_ < 0.05). Subgroup analysis showed that there was an interaction between CRP and smoking status on risk for overall cancer, lung, uterus, and prostate cancer (Additional file 2: Table S12). Besides, findings from sensitivity analyses were almost consistent with the primary analysis (Additional file 2: Table S13, S14, S15 and S17). The associations of CRP and cancer risks became stronger by only including patients that were diagnosed within 2 years of follow-up, indicating the associations were true (Additional file 2: Table S16).

**Table 3:** Hazard ratios (HRs) for cancer outcomes among three CRP groups based on observation data (compared with low CRP (≤1 mg/L) group, the hazard ratio for cancer risk among average/high CRP groups (per 1 mg/L higher CRP concentration))

	Low CRP (<1 mg/L)	Average CRP (1 to 3 mg/L)				High CRP (>3 mg/L)			FDR-adjusted ***P***_**trend**_
	No. (incident cases)	No. (incident cases)	HR (95%CI)	***P***		No (incident cases)	HR (95%CI)	***P***	
**Overall cancer**	166,888 (12,380)	160,143 (13,793)	1.05 (1.02, 1.07)	0.001	<0.001	93,933 (8806)	1.15 (1.12, 1.18)	<0.001	<0.001
**Head & neck (C00–14)**	154,674 (166)	146,535 (185)	1.06 (0.85, 1.31)	0.617	0.006	85,283 (156)	1.46 (1.15, 1.85)	0.002	0.002
**Esophagus (C15)**	154,643 (136)	146,558 (208)	1.20 (0.96, 1.49)	0.115	0.064	85,273 (146)	1.31 (1.01, 1.68)	0.038	0.038
**Stomach (C16)**	154,605 (97)	146,482 (132)	1.10 (0.84, 1.44)	0.500	0.064	85,232 (106)	1.38 (1.02, 1.87)	0.034	0.035
**Colorectal (C18–20)**	155,467 (959)	147,536 (1186)	1.11 (1.02, 1.21)	0.019	<0.001	85,917 (791)	1.27 (1.14, 1.40)	<0.001	<0.001
**Liver (C22)**	154,584 (76)	146,453 (103)	1.10 (0.81, 1.50)	0.523	0.003	85,230 (103)	1.73 (1.25, 2.40)	0.001	0.001
**Gallbladder (C23–24)**	154,553 (45)	146,399 (49)	0.82 (0.54, 1.24)	0.344	0.818	85,172 (45)	1.06 (0.67, 1.68)	0.805	0.818
**Pancreas (C25)**	154,678 (170)	146,590 (240)	1.15 (0.94, 1.41)	0.164	0.212	85,286 (159)	1.18 (0.93, 1.50)	0.162	0.154
**Lung (C33–34)**	154,927 (419)	147,068 (718)	1.37 (1.21, 1.55)	<0.001	<0.001	85,882 (755)	2.08 (1.83, 2.36)	<0.001	<0.001
**Melanoma (C43)**	155,064 (559)	146,902 (552)	1.01 (0.89,1.14)	0.936	0.792	85,437 (310)	1.03 (0.89, 1.21)	0.661	0.684
**Non-melanotic skin (C44)**	158,800 (4294)	150,650 (4,303)	0.99 (0.95,1.04)	0.743	0.818	87,430 (2305)	0.99 (0.94, 1.05)	0.814	0.785
**Breast (C50)**	83,335 (1704)	77,468 (1940)	1.15 (1.08, 1.23)	<0.001	<0.001	51,446 (1371)	1.20 (1.11, 1.31)	<0.001	<0.001
**Uterus (C54–55)**	81,841 (210)	75,810 (281)	1.01 (0.84, 1.21)	0.957	0.116	50,382 (306)	1.20 (0.97, 1.47)	0.087	0.074
**Ovary (C56)**	81,804 (173)	75,744 (215)	1.27 (1.03, 1.56)	0.028	0.024	50,223 (147)	1.37 (1.07, 1.76)	0.014	0.011
**Prostate (C61)**	74,992 (2116)	73,042 (2221)	0.99 (0.93, 1.06)	0.832	0.221	36,092 (1043)	0.94 (0.87, 1.02)	0.134	0.171
**Kidney (C64)**	154,689 (181)	146,631 (282)	1.26 (1.04, 1.53)	0.018	<0.001	85,362 (235)	1.64 (1.33, 2.03)	<0.001	<0.001
**Bladder (C67)**	154,689 (181)	146,581 (231)	1.01 (0.83, 1.23)	0.929	0.562	85,281 (154)	1.09 (0.87, 1.38)	0.447	0.460
**CNS (C70–72)**	154,662 (154)	146,523 (173)	1.11 (0.89, 1.40)	0.346	0.185	85,232 (105)	1.23 (0.94, 1.62)	0.129	0.126
**Thyroid (C73)**	154,590 (82)	146,441 (91)	1.09 (0.80, 1.49)	0.596	0.818	85,179 (52)	0.93 (0.63, 1.38)	0.711	0.789
**Non-Hodgkin lymphoma (C82–85, 96)**	154,862 (354)	146,772 (422)	1.14 (0.99, 1.32)	0.078	<0.001	85,447 (320)	1.53 (1.30, 1.81)	<0.001	<0.001
**Multiple myeloma (C90)**	154,670 (163)	146,502 (152)	0.82 (0.65, 1.03)	0.082	0.058	85,211 (84)	0.74 (0.55, 0.98)	0.039	0.029
**CLL (C91)**	154,675 (167)	146,491 (141)	0.74 (0.58, 0.93)	0.010	0.015	85,205 (78)	0.69 (0.51, 0.92)	0.012	0.006

Adjusted for age, sex (female, male), ethnic (White, Asian, African, mixed background, unknown), education (no degree, degree, unknown), Townsend deprivation index, standing height, BMI, smoking status (never, previous, current, unknown), alcohol use (never, previous, current, unknown), physical activity (<600 MET/week, 600–3000 MET/week, ≥3000 MET/week), family cancer (no, yes), and assessment center. Additionally adjusted for menopausal (no, yes, not sure, unknown), oral contraceptive use (never, ever, unknown), hormone replacement therapy (never, ever, unknown) for female. *CNS*, central nervous system; *CLL*, chronic lymphocytic leukemia

### Mendelian randomization analyses

**Fig. 2:**
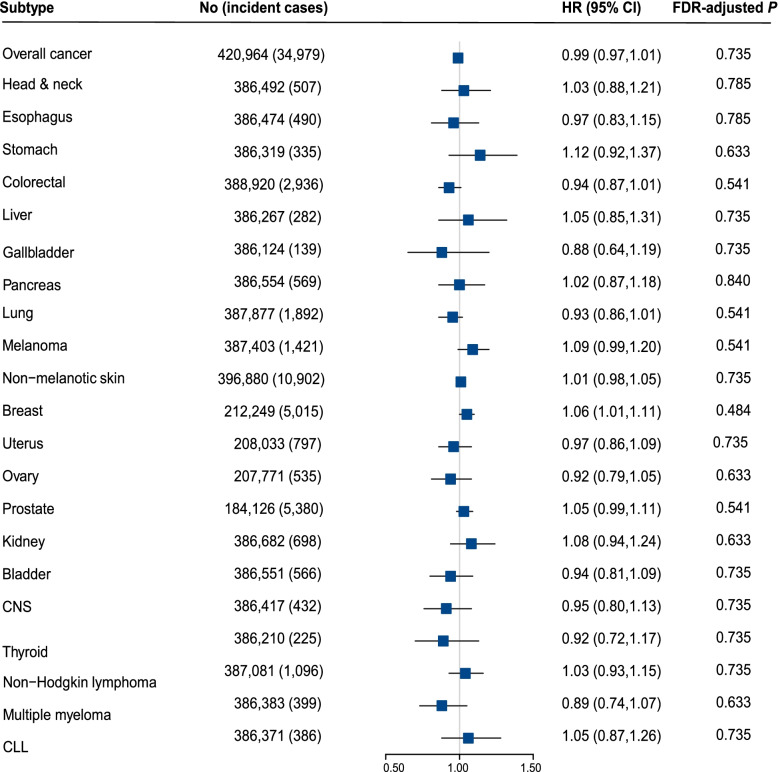
The casual relationship between the CRP and cancer outcomes using linear MR analysis. Adjusted for age, sex (female, male), BMI, smoking status (never, previous, current, unknown), and top ten genetic principal components, and chip. CNS, central nervous system; CLL, chronic lymphocytic leukemia

**Fig. 3:**
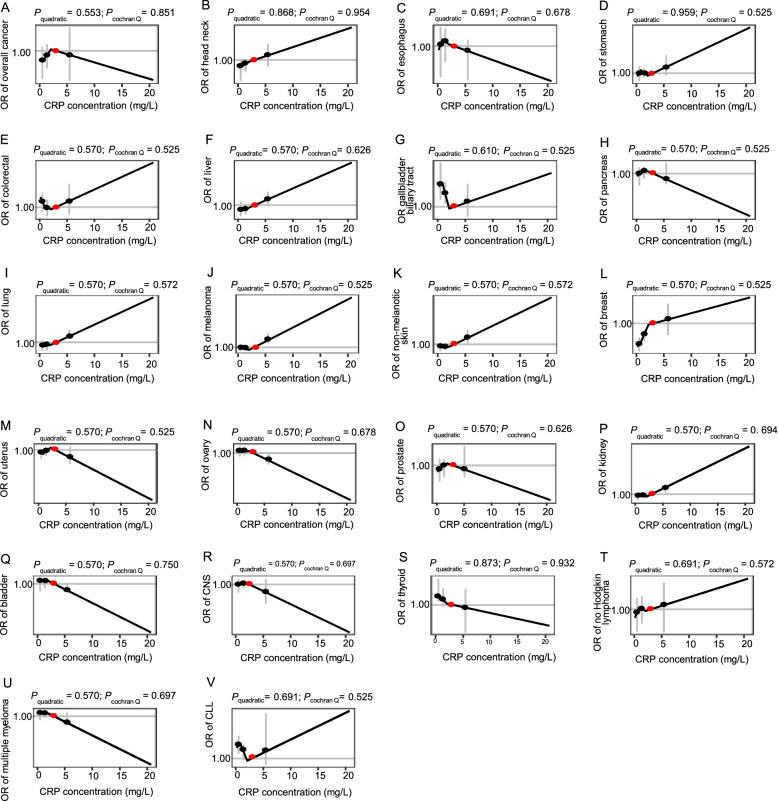
Shape of casual relationship between CRP and cancer outcomes using the piecewise linear non-linear MR method. All *P* values in the figure are false-discovery rate adjusted *P* values. Adjusted for age, sex (female, male), BMI, smoking status (never, previous, current, unknown), top ten principal components of ancestry, and genotyping batch. CNS, central nervous system; CLL, chronic lymphocytic leukemia

As shown in Fig. 2, there were no linear associations between genetically predicted CRP concentration and risk of overall cancer, as well as site-specific cancer (FDR-adjusted *P* > 0.05). Besides, similar results were also observed in the sensitivity analyses of re-estimating the association between log-transformed CRP concentration and cancer risks (Additional file 3: Figure S3), re-evaluating in individuals of genetically confirmed British ancestry (Additional file 3: Figure S4), or using rs2794520 as an instrument variable (Additional file 3: Figure S5). Furthermore, no evidences of non-linear causal effects were observed between genetically predicted CRP concentration and risk of overall cancer or site-specific cancer (Fig. 3).

## Discussion

The role of inflammation in tumorigenesis is now generally accepted, but in which a direct causal relationship has yet not been proven [25]. In the present study, based on a large-scale prospective cohort study, we found elevated CRP concentration at baseline were associated with increased risk of incident cancer events during the follow-up. For site-specific cancer, positive associations were also observed for cancers of head and neck, esophagus, stomach, colorectal, liver, lung, breast, kidney cancer, and non-Hodgkin lymphoma, while negative association was observed for CLL. Although we observed three different patterns of non-linear associations between CRP concentration and site-specific cancers risks, obvious inflection points were observed at 3mg/L, and 1mg/L after log-transformed, which were in consistent with the findings for cardiovascular disease [24]. However, both linear and non-linear MR analysis demonstrated no associations between genetically predicted CRP and the risk of cancer.

Thus far, several hypotheses have been proposed to explain the potential mechanisms of the associations between elevated CRP concentration and risk of cancer: (1) tumor tissue would cause inflammation and thus increase serum level of CRP; (2) tumor cells could produce various cytokines and chemokines that stimulate CRP production in the liver; (3) CRP was a part of a host immune response to tumor cells; (4) CRP is a marker of chronic inflammation, which would promote carcinogenesis by creating an attractive environment; and (5) CRP could be acted as an internal exposure marker, which reflected the aging state of the body [26–28].

Inconsistent associations have been reported between CRP and cancer risk in previous prospective cohort studies [29–31]. Our results were in consistent with that from Danish cohort study and EPIC-Heidelberg Study, which demonstrated associations for overall cancer, lung cancer, and colorectal cancer [26, 27]. Moreover, our research further extended the positive associations to cancers of head and neck, liver, kidney, and non-Hodgkin lymphoma. Although CRP had been reported as a poor prognostic factor of CLL [32, 33], our results identified negative associations between CRP and incident cancer events of CLL during the follow-up, which might indicate preclinical patients had been undergoing immune dysfunction [34]. No associations have been reported for the associations between CRP concentration and breast cancer [35–37], which were consistent with our tests of linear associations. Interestingly, our results further demonstrated a non-linear “increase-to-decrease” association pattern between CRP concentration and the breast cancer, which showed statistically significant heterogeneities for the associations between low (≤ 3mg/L) and high (> 3mg/L) CRP groups. The biological mechanisms underlying this special association pattern remain to be further studied.

In risk stratification of cardiovascular disease, the groups of low risk (< 1.0 mg/L), average risk (1.0 to 3.0 mg/L), and high risk (> 3.0 mg/L) approximately correspond to tertiles of CRP in the general adult population, which were derived from more than 40,000 persons in more than 15 populations [24]. In our study, we also observed an inflection point of the association between CRP and cancer risk at 3mg/L in different non-linear association patterns. After log-transformed, we also observed an inflection point at 1mg/L. This was probably because log-transformed can get a higher resolution for data with less than 1, but lower resolution for data with lager than 1. Unsurprisingly, the association pattern after log-transformed was obviously different from that of initial CRP level (Additional file 3: Figure S1 and Figure S2), this was probably because the meaning of the *X*-axis has been changed after log-transformed (i.e., 1 corresponding to 2.7mg/L, while 2 corresponding to 7.4 mg/L). It was especially worth noting that the increase of risk is more pronounced among individuals of low CRP group (<3mg/L), which highlighted the important role of chronic low-grade inflammation in carcinogenesis [28].

Although observational analysis showed significant associations between CRP concentration and incident cancer risk in the prospective cohort, MR analysis did not support the causal relationship. Similar discrepancy between the observational and MR analyses had been reported for breast cancer, colorectal cancer, prostate cancer, and lung cancer [8, 11, 12, 38]. In the observational analysis, potential confounding factors have been fully adjusted in the main and sensitivity analysis; moreover, similar associations were observed after excluding patients diagnosed within 2 years of follow-up. This indicated the discrepancy was not likely to be biased by confounding or reverse causality. Therefore, we hold the opinion that the CRP was probably not the cause of cancer itself, but acted as a response marker of environmental risk factors (i.e., smoking, air pollutions, and aging), which can cause chronic low-grade inflammation. However, this needed to be further evaluated as only 2.6% of the variance of CRP can be explained by the genetic instrumental variable.

The main advantage of this study lies in a comprehensive analysis, including both observational analysis and MR analysis, of the associations between CRP and overall cancer risk, as well as site-specific cancer. However, we also acknowledge several limitations of this study. First, CRP concentration was assessed on a single measure at baseline, and changes during the follow-up may have an effect on risk evaluation. Second, although there was a strong association between genetic instrumental variable and CRP concentration, these variants only explain a small proportion of variance in CRP concentration. Third, CRP is only one of the inflammatory markers, and a recent study has reported that genetically predicted circulating concentrations of several inflammatory-related cytokines were associated with the risk of breast, endometrial, lung, ovarian, and prostate cancer [39]. Fourth, evidences have indicated the poor representativeness of subjects in UK Biobank because of low participation and healthy volunteer bias; therefore, further studies are warranted to evaluate to what degree our findings may be generalized to the general population [40]. Fifth, most of the participants were of British ancestry in our study, which may limit the generalizability of our findings to other populations. Hence, these findings should be further validated in more diverse populations.

## Conclusions

Based on a large-scale prospective cohort study, we demonstrated a positive association between CRP concentration and overall cancer risk, as well as nine site-specific cancers. Although we observed three non-linear association patterns between CRP concentration and site-specific cancers, the inflection points were at 3mg/L of CRP concentration, and at 1mg/L after log-transformed, consistently. However, no causal relationship was observed in the linear and non-linear MR analysis. These findings collectively indicated that CRP was a potential biomarker for cancer risk stratification, which might also take 1mg/L and 3mg/L as cutoff points.

## Supplementary Information

**Additional file 1.** Supplemental methods.

**Additional file 2: Table S1.** Diagnosis of cancer based on ICD10. **Table S2.** The percent of missing values for covariates. **Table S3.** Five SNPs associated with both colorectal cancer and serum CRP concentration. **Table S4.** Observational association between CRP and cancer risks in total sample. **Table S5.** Univariable analysis of the observational association between the CRP and cancer risk. **Table S6.** Observational association between the log-transformed CRP concentration and cancer risks. **Table S7.** Sensitivity analysis of the observational association between the CRP and cancer outcomes by additionally adjusting for cardiovascular and diabetes diseases. **Table S8.** Sensitivity analysis of the observational association between the CRP and cancer outcomes by additionally adjusting for drugs. **Table S9.** Sensitivity analysis of the observational association between the CRP and cancer outcomes by excluding of patients diagnosed in the first two follow-up. **Table S10.** Sensitivity analysis of the observational association between the CRP and cancer outcomes by only including of patients diagnosed in the first two follow-up. **Table S11.** Sensitivity analysis of the observational association between the CRP and cancer outcomes by excluding of participants with CRP > 10 mg/L. **Table S12.** Subgroup analysis of association between CRP and cancer risk. **Table S13.** Hazard ratios for cancer outcomes among three CRP groups based on observation data by additionally adjusting for cardiovascular disease and diabetes. **Table S14.** Hazard ratios for cancer outcomes among three CRP groups based on observation data by additionally adjusting for drugs. **Table S15.** Hazard ratios for cancer outcomes among three CRP groups based on observation data by excluding of patients diagnosed in the first two follow-up. **Table S16.** Hazard ratios for cancer outcomes among three CRP groups based on observation data by only including of patients diagnosed in the first two follow-up. **Table S17.** Hazard ratios for cancer outcomes among three CRP groups based on observation data by excluding of participants with CRP > 10 mg/L.

**Additional file 3: Figure S1.** Analysis of the shape of the relationship between CRP and cancer outcomes by excluding of patients diagnosed in the first two follow-up. **Figure S2.** Analysis of the shape of the relationship between log-transformed CRP concentration and cancer outcomes. **Figure S3.** Sensitivity analysis of the linear MR analysis of log-transformed CRP and cancer risks. **Figure S4.** Sensitivity analysis of the linear MR analysis in people genetically confirmed of British ancestry. **Figure S5.** Sensitivity analysis of the linear MR analysis using rs2794520 as instrument variable.

## Data Availability

Data are, however, available from the authors upon reasonable request and with permission of UK Biobank.

## Abbreviations

AIC: Akaike information criterion
BMI: Body mass index
CNS: Central nervous system
CLL: Chronic lymphocytic leukemia
CRP: C-reactive protein
FDR: False-discovery rate
HR: Hazard ratio
IQR: Interquartile range
LACE: Localized average causal effect
MR: Mendelian randomization
SNPs: Single nucleotide polymorphisms
wGRS: Weighted genetic risk score
