# Supplementary material for: C-reactive protein and cancer risk: a pan-cancer study of prospective cohort and Mendelian randomization analysis
Source: BMC Med. 2022 Sep 19;20:301. doi: 10.1186/s12916-022-02506-x (PMC9484145; doi:10.1186/s12916-022-02506-x)
Supplement: Supplementary file 1 — Additional file 1. Supplemental methods. [file 12916_2022_2506_MOESM1_ESM.doc]

***Supplemental methods***

***Genetic instrument for serum CRP levels***

The weighted genetic risk score (wGRS) for each individual was calculated by multiplying the number of risk alleles and weights:


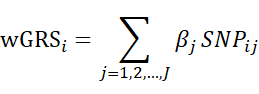


where j indexes the SNPs, and β represents the weight value reported by the previous GWAS.

The F statistic was calculated to test the statistical significance of the correlation between the instrument variables and CRP:


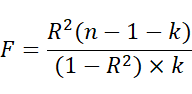


where *R^2^* represents the variability explained by instrumental variable (*R^2^*=0.026), *n* indexes the number of individuals (n=420,964), and *k* refers to the number of SNPs (*k*=52). Cumulatively, the *F*-statistic was 216, which indicates a strong relationship between instrumental variable and CRP.
