# Supplementary material for: C-reactive protein and cancer risk: a pan-cancer study of prospective cohort and Mendelian randomization analysis
Source: BMC Med. 2022 Sep 19;20:301. doi: 10.1186/s12916-022-02506-x (PMC9484145; doi:10.1186/s12916-022-02506-x)
Supplement: Supplementary file 2 — Additional file 2: Table S1. Diagnosis of cancer based on ICD10. Table S2. The percent of missing values for covariates. Table S3. Five SNPs associated with both colorectal cancer and serum CRP concentration. Table S4. Observational association between CRP and cancer risks in total sample. Table S5. Univariable analysis of the observational association between the CRP and cancer risk. Table S6. Observational association between the log-transformed CRP concentration and cancer risks. Table S7. Sensitivity analysis of the observational association between the CRP and cancer outcomes by additionally adjusting for cardiovascular and diabetes diseases. Table S8. Sensitivity analysis of the observational association between the CRP and cancer outcomes by additionally adjusting for drugs. Table S9. Sensitivity analysis of the observational association between the CRP and cancer outcomes by excluding of patients diagnosed in the first two follow-up. Table S10. Sensitivity analysis of the observational association between the CRP and cancer outcomes by only including of patients diagnosed in the first two follow-up. Table S11. Sensitivity analysis of the observational association between the CRP and cancer outcomes by excluding of participants with CRP > 10 mg/L. Table S12. Subgroup analysis of association between CRP and cancer risk. Table S13. Hazard ratios for cancer outcomes among three CRP groups based on observation data by additionally adjusting for cardiovascular disease and diabetes. Table S14. Hazard ratios for cancer outcomes among three CRP groups based on observation data by additionally adjusting for drugs. Table S15. Hazard ratios for cancer outcomes among three CRP groups based on observation data by excluding of patients diagnosed in the first two follow-up. Table S16. Hazard ratios for cancer outcomes among three CRP groups based on observation data by only including of patients diagnosed in the first two follow-up. Table S17. Hazard ratios for cancer outco [file 12916_2022_2506_MOESM2_ESM.doc]

***Supplemental results***

***Table S1. Diagnosis of cancer based on ICD10***

| **index** | **Cancer site** | **ICD10** |
| --- | --- | --- |
| 1 | Head and neck | C00-C14 |
| 2 | Esophagus | C15 |
| 3 | Stomach | C16 |
| 4 | Colorectal | C18-C20 |
| 5 | Liver | C22 |
| 6 | Gallbladder | C23-24 |
| 7 | Pancreas | C25 |
| 8 | Lung | C33-C34 |
| 9 | Melanoma | C43 |
| 10 | Non-melanotic skin | C44 |
| 11 | Breast | C50 |
| 12 | Uterus | C54-C55 |
| 13 | Ovary | C56 |
| 14 | Prostate | C61 |
| 15 | Kidney | C64 |
| 16 | Bladder | C67 |
| 17 | CNS | C70-C72 |
| 18 | Thyroid | C73 |
| 19 | Non-Hodgkin lymphoma | C82-85,96 |
| 20 | Multiple Myeloma | C90 |
| 21 | CLL | C91 |

***Table S2.*** *The percent of missing values for covariates*

|  | **Number** | **Percent (%)** |
| --- | --- | --- |
| **Age at baseline** | 0 | 0.0 |
| **Sex** | 0 | 0.0 |
| **Ethnic** | 5,940 | 1.4 |
| **Education** | 5,026 | 1.2 |
| **Townsend deprivation index** | 526 | 0.1 |
| **Standing height** | 1,236 | 0.3 |
| **BMI** | 1,670 | 0.4 |
| **Smoking status** | 2,118 | 0.5 |
| **Alcohol use** | 1,078 | 0.3 |
| **Physical activity** **MET/week** | 80,237 | 0.3 |
| **Having Family cancer history** | 0 | 0.0 |
| **Menopausal** | 687 | 0.3 |
| **O****ral contraceptive use** | 1,039 | 0.5 |
| **Hormone replacement therapy** | 1,175 | 0.5 |
| **Assessment Centre** | 0 | 0.0 |

**Table S3.** Five SNPs associated with both colorectal cancer and serum CRP concentration

| **SNP** | **Chromosome** | **Position** | **Effect allele** | **Beta** | **Se** | ***P*** |
| --- | --- | --- | --- | --- | --- | --- |
| **rs2064009** | 8 | 117007850 | T | -0.073 | 0.026 | 0.005 |
| **rs4420638** | 19 | 45422946 | A | -0.089 | 0.033 | 0.007 |
| **rs11108056** | 11 | 95855385 | C | -0.058 | 0.027 | 0.029 |
| **rs10925027** | 1 | 247612562 | T | -0.054 | 0.027 | 0.041 |
| **rs10521222** | 16 | 51158710 | C | -0.115 | 0.058 | 0.048 |

**Table S4.** Observational association between CRP and cancer risks in total sample

|  | **No (incident cases)** | **Person years** | **Per 1 mg/L increase in CRP** |  | **Per 1 SD increase in CRP** |  | ***P*** |
| --- | --- | --- | --- | --- | --- | --- | --- |
|  |  |  | **HR (95%CI)** |  | **HR (95%CI)** |  |  |
| **Overall cancer** | 420,964 (34,979) | 2,873,325 | 1.02 (1.01,1.02) |  | 1.05 (1.04,1.06) |  | <0.001 |
| **Head & neck (C00-14)** | 386,492 (507) | 2,741,258 | 1.04 (1.02,1.06) |  | 1.14 (1.06,1.22) |  | <0.001 |
| **Esophagus (C15)** | 386,474 (490) | 2,741,176 | 1.04 (1.01,1.06) |  | 1.12 (1.04,1.20) |  | 0.002 |
| **Stomach (C16)** | 386,319 (335) | 2,740,534 | 1.03 (1.01,1.06) |  | 1.12 (1.02,1.22) |  | 0.012 |
| **Colorectal (C18-20)** | 388,920 (2,936) | 2,750,426 | 1.02 (1.01,1.03) |  | 1.08 (1.05,1.12) |  | <0.001 |
| **Liver (C22)** | 386,267 (282) | 2,740,446 | 1.04 (1.01,1.07) |  | 1.15 (1.05,1.26) |  | 0.003 |
| **Gallbladder (C23-24)** | 386,124 (139) | 2,739,827 | 0.97 (0.91,1.02) |  | 0.90 (0.74,1.08) |  | 0.247 |
| **Pancreas (C25)** | 386,554 (569) | 2,741,611 | 1.00 (0.98,1.03) |  | 1.01 (0.94,1.10) |  | 0.725 |
| **Lung (C33-34)** | 387,877 (1,892) | 2,746,976 | 1.06 (1.05,1.07) |  | 1.21 (1.17,1.25) |  | <0.001 |
| **Melanoma (C43)** | 387,403 (1,421) | 2,744,818 | 1.01 (0.99,1.03) |  | 1.03 (0.98,1.09) |  | 0.289 |
| **Non-melanotic skin (C44)** | 396,880 (10,902) | 2,782,977 | 1.00 (1.00,1.01) |  | 1.01 (0.99,1.03) |  | 0.261 |
| **Breast (C50)** | 212,249 (5,015) | 1,492,129 | 1.00 (1.00,1.01) |  | 1.02 (0.99,1.05) |  | 0.270 |
| **Uterus (C54-55)** | 208,033 (797) | 1,476,462 | 1.02 (1.00,1.04) |  | 1.08 (1.02,1.15) |  | 0.013 |
| **Ovary (C56)** | 207,771 (535) | 1,475,432 | 1.02 (0.99,1.04) |  | 1.06 (0.97,1.15) |  | 0.180 |
| **Prostate (C61)** | 184,126 (5,380) | 1,286,416 | 0.99 (0.99,1.00) |  | 0.98 (0.96,1.01) |  | 0.200 |
| **Kidney (C64)** | 386,682 (698) | 2,742,036 | 1.05 (1.04,1.07) |  | 1.19 (1.13,1.26) |  | <0.001 |
| **Bladder (C67)** | 386,551 (566) | 2,741,376 | 1.01 (0.99,1.04) |  | 1.05 (0.97,1.13) |  | 0.213 |
| **CNS (C70-72)** | 386,417 (432) | 2,740,957 | 1.00 (0.97,1.03) |  | 1.00 (0.90,1.10) |  | 0.989 |
| **Thyroid (C73)** | 386,210 (225) | 2,740,111 | 1.00 (0.96,1.04) |  | 1.00 (0.87,1.14) |  | 0.945 |
| **Non-Hodgkin lymphoma (C82-85,96)** | 387,081 (1,096) | 2,743,484 | 1.04 (1.03,1.06) |  | 1.14 (1.09,1.20) |  | <0.001 |
| **Multiple myeloma (C90)** | 386,383 (399) | 2,740,916 | 0.96 (0.93,1.00) |  | 0.88 (0.77,0.99) |  | 0.035 |
| **CLL (C91)** | 386,371 (386) | 2,740,738 | 0.97 (0.94,1.01) |  | 0.91 (0.81,1.03) |  | 0.141 |

Adjusted for age, sex (female, male), ethnic (White, Asian, African, mixed background, unknown), education (no degree, degree, unknown), Townsend deprivation index, standing height, BMI, smoking status (never, previous, current, unknown), alcohol use (never, previous, current, unknown), physical activity (<600 MET/week, 600-3,000 MET/week, ≥3,000 MET/week), family cancer (no, yes), and assessment centre. Additionally adjusted for menopausal (no, yes, not sure, unknown), oral contraceptive use (never, ever, unknown), hormone replacement therapy (never, ever, unknown) for female. CNS, central nervous system; CLL, chronic lymphocytic leukemia.

**Table S5.** Univariable analysis of the observational association between the CRP and cancer risk^*^

|  | **≤ 3mg/L** | | | |  | **>3 mg/L** | | | | |
| --- | --- | --- | --- | --- | --- | --- | --- | --- | --- | --- |
|  | **No**  **(incident cases)** | **Person years** | **HR (95%CI)** | ***P*** |  | **No**  **(incident cases)** | **Person years** | **HR (95%CI)** | ***P*** | ***P* _heterogeneity_** |
| **Overall cancer** | 327,031 (26,173) | 2,237,479 | 1.12 (1.11,1.14) | <0.001 |  | 93,933 (8,806) | 635,846 | 1.01 (1.01,1.02) | <0.001 | <0.001 |
| **Head & neck (C00-14)** | 301,209 (351) | 2,137,461 | 1.15 (1.01,1.32) | 0.041 |  | 85,283 (156) | 603,797 | 1.03 (0.99,1.06) | 0.106 | 0.102 |
| **Esophagus (C15)** | 301,201(344) | 2,137,476 | 1.43 (1.26,1.64) | <0.001 |  | 85,273 (146) | 603,701 | 1.04 (1.01,1.07) | 0.006 | <0.001 |
| **Stomach (C16)** | 301,087 (229) | 2,136,975 | 1.26 (1.06,1.48) | 0.007 |  | 85,232 (106) | 603,559 | 1.03 (0.99,1.07) | 0.118 | 0.023 |
| **Colorectal (C18-20)** | 303,003 (2,145) | 2,144,527 | 1.23 (1.17,1.30) | <0.001 |  | 85,917 (791) | 605,899 | 1.02 (1.00,1.03) | 0.022 | <0.001 |
| **Liver (C22)** | 301,037 (179) | 2,136,852 | 1.45 (1.20,1.74) | <0.001 |  | 85,230 (103) | 603,594 | 1.01 (0.97,1.05) | 0.663 | <0.001 |
| **Gallbladder (C23-24)** | 300,952 (94) | 2,136,473 | 1.15 (0.88,1.50) | 0.300 |  | 85,172 (45) | 603,354 | 0.91 (0.83,1.00) | 0.055 | 0.108 |
| **Pancreas (C25)** | 301,268 (410) | 2,137,813 | 1.33 (1.18,1.50) | <0.001 |  | 85,286 (159) | 603,798 | 0.99 (0.96,1.03) | 0.673 | <0.001 |
| **Lung (C33-34)** | 301,995 (1,137) | 2,140,855 | 1.52 (1.42,1.64) | <0.001 |  | 85,882 (755) | 606,121 | 1.04 (1.03,1.06) | <0.001 | <0.001 |
| **Melanoma (C43)** | 301,966 (1,111) | 2,140,358 | 1.02 (0.94,1.10) | 0.673 |  | 85,437 (310) | 604,460 | 1.01 (0.99,1.03) | 0.357 | 0.884 |
| **Non-melanotic skin (C44)** | 309,450 (8,597) | 2,170,575 | 1.05 (1.02,1.08) | 0.002 |  | 87,430 (2,305) | 612,401 | 1.01 (1.00,1.01) | 0.163 | 0.009 |
| **Breast (C50)** | 160,803 (3,644) | 1,130,896 | 1.14 (1.09,1.18) | <0.001 |  | 51,446 (1,371) | 361,233 | 0.99 (0.98,1.00) | 0.087 | <0.001 |
| **Uterus (C54-55)** | 157,651 (491) | 1,119,136 | 1.41 (1.26,1.57) | <0.001 |  | 50,382 (306) | 357,326 | 1.03 (1.01,1.05) | 0.006 | <0.001 |
| **Ovary (C56)** | 157,548 (388) | 1,118,715 | 1.17 (1.03,1.33) | 0.015 |  | 50,223 (147) | 356,717 | 1.00 (0.96,1.03) | 0.925 | 0.018 |
| **Prostate (C61)** | 148,034 (4,337) | 1,035,523 | 1.05 (1.01,1.09) | 0.015 |  | 36,092 (1,043) | 250,894 | 1.00 (0.99,1.02) | 0.703 | 0.028 |
| **Kidney (C64)** | 301,320 (463) | 2,137,990 | 1.39 (1.24,1.56) | <0.001 |  | 85,362 (235) | 604,047 | 1.05 (1.03,1.08) | 0.000 | <0.001 |
| **Bladder (C67)** | 301,270 (412) | 2,137,591 | 1.27 (1.12,1.44) | <0.001 |  | 85,281 (154) | 603,785 | 1.02 (0.98,1.05) | 0.330 | 0.001 |
| **CNS (C70-72)** | 301,185 (327) | 2,137,370 | 1.17 (1.01,1.35) | 0.032 |  | 85,232 (105) | 603,587 | 0.96 (0.91,1.01) | 0.099 | 0.010 |
| **Thyroid (C73)** | 301,031 (173) | 2,136,726 | 1.16 (0.95,1.40) | 0.147 |  | 85,179 (52) | 603,385 | 1.02 (0.96,1.07) | 0.579 | 0.214 |
| **Non-Hodgkin lymphoma**  **(C82-85,96)** | 301,634 (776) | 2,139,208 | 1.16 (1.06,1.27) | 0.002 |  | 85,447 (320) | 604,276 | 1.02 (1.00,1.05) | 0.046 | 0.010 |
| **Multiple myeloma (C90)** | 301,172 (315) | 2,137,379 | 1.05 (0.91,1.22) | 0.515 |  | 85,211 (84) | 603,538 | 0.96 (0.91,1.02) | 0.167 | 0.276 |
| **CLL (C91)** | 301,166 (308) | 2,137,248 | 0.94 (0.80,1.09) | 0.421 |  | 85,205 (78) | 603,491 | 1.00 (0.95,1.05) | 0.946 | 0.430 |

^*^Hazard ratios for cancer outcomes per 1 mg/L higher CRP

**Table S6. Observational association between the log-transformed CRP concentration and cancer risks**

|  | **≤ 3mg/L** | | | |  | **>3 mg/L** | | | | |
| --- | --- | --- | --- | --- | --- | --- | --- | --- | --- | --- |
|  | **No**  **(incident cases)** | **Person years** | **HR (95%CI)** | ***P*** |  | **No**  **(incident cases)** | **Person years** | **HR (95%CI)** | ***P*** | ***P* _heterogeneity_** |
| **Overall cancer** | 327,031 (26,173) | 2,237,479 | 1.04 (1.03,1.06) | <0.001 |  | 93,933 (8,806) | 635,846 | 1.10 (1.06,1.14) | <0.001 | <0.001 |
| **Head & neck (C00-14)** | 301,209 (351) | 2,137,461 | 1.08 (0.92,1.27) | 0.344 |  | 85,283 (156) | 603,797 | 1.23 (0.93,1.62) | 0.141 | 0.402 |
| **Esophagus (C15)** | 301,201 (344) | 2,137,476 | 1.14 (0.96,1.35) | 0.131 |  | 85,273 (146) | 603,701 | 1.35 (1.02,1.78) | 0.035 | 0.310 |
| **Stomach (C16)** | 301,087 (229) | 2,136,975 | 1.03 (0.84,1.26) | 0.786 |  | 85,232 (106) | 603,559 | 1.40 (1.00,1.95) | 0.047 | 0.022 |
| **Colorectal (C18-20)** | 303,003 (2,145) | 2,144,527 | 1.12 (1.05,1.20) | 0.001 |  | 85,917 (791) | 605,899 | 1.15 (1.01,1.30) | 0.034 | 0.062 |
| **Liver (C22)** | 301,037 (179) | 2,136,852 | 1.15 (0.92,1.46) | 0.223 |  | 85,230 (103) | 603,594 | 1.05 (0.74,1.49) | 0.783 | 0.419 |
| **Gallbladder (C23-24)** | 300,952 (94) | 2,136,473 | 0.89 (0.65,1.21) | 0.446 |  | 85,172 (45) | 603,354 | 0.44 (0.22,0.86) | 0.017 | 0.001 |
| **Pancreas (C25)** | 301,268 (410) | 2,137,813 | 1.16 (0.99,1.35) | 0.060 |  | 85,286 (159) | 603,798 | 0.89 (0.66,1.20) | 0.447 | 0.304 |
| **Lung (C33-34)** | 301,995 (1,137) | 2,140,855 | 1.28 (1.17,1.40) | <0.001 |  | 85,882 (755) | 606,121 | 1.40 (1.24,1.58) | <0.001 | <0.001 |
| **Melanoma (C43)** | 301,966 (1,111) | 2,140,358 | 0.98 (0.90,1.07) | 0.677 |  | 85,437 (310) | 604,460 | 1.16 (0.95,1.42) | 0.136 | <0.001 |
| **Non-melanotic skin (C44)** | 309,450 (8,597) | 2,170,575 | 1.00 (0.97,1.03) | 0.960 |  | 87,430 (2,305) | 612,401 | 1.06 (0.98,1.14) | 0.136 | <0.001 |
| **Breast (C50)** | 160,803 (3,644) | 1,130,896 | 1.09 (1.04,1.14) | 0.001 |  | 51,446 (1,371) | 361,233 | 0.91 (0.82,1.01) | 0.085 | 0.117 |
| **Uterus (C54-55)** | 157,651 (491) | 1,119,136 | 1.10 (0.96,1.26) | 0.187 |  | 50,382 (306) | 357,326 | 1.10 (0.89,1.36) | 0.362 | 0.485 |
| **Ovary (C56)** | 157,548 (388) | 1,118,715 | 1.09 (0.93,1.26) | 0.289 |  | 50,223 (147) | 356,717 | 0.99 (0.73,1.35) | 0.959 | 0.089 |
| **Prostate (C61)** | 148,034 (4,337) | 1,035,523 | 1.00 (0.96,1.05) | 0.901 |  | 36,092 (1,043) | 250,894 | 1.00 (0.90,1.12) | 0.930 | <0.001 |
| **Kidney (C64)** | 301,320 (463) | 2,137,990 | 1.16 (1.00,1.34) | 0.052 |  | 85,362 (235) | 604,047 | 1.60 (1.29,1.98) | <0.001 | 0.002 |
| **Bladder (C67)** | 301,270 (412) | 2,137,591 | 1.04 (0.90,1.21) | 0.574 |  | 85,281 (154) | 603,785 | 1.09 (0.82,1.45) | 0.561 | 0.003 |
| **CNS (C70-72)** | 301,185 (327) | 2,137,370 | 1.17 (0.99,1.38) | 0.064 |  | 85,232 (105) | 603,587 | 0.72 (0.49,1.06) | 0.100 | 0.071 |
| **Thyroid (C73)** | 301,031 (173) | 2,136,726 | 1.05 (0.84,1.32) | 0.644 |  | 85,179 (52) | 603,385 | 1.26 (0.77,2.05) | 0.351 | 0.133 |
| **Non-Hodgkin lymphoma (C82-85,96)** | 301,634 (776) | 2,139,208 | 1.09 (0.98,1.21) | 0.132 |  | 85,447 (320) | 604,276 | 1.25 (1.03,1.52) | 0.023 | 0.416 |
| **Multiple myeloma (C90)** | 301,172 (315) | 2,137,379 | 0.88 (0.74,1.04) | 0.134 |  | 85,211 (84) | 603,538 | 0.66 (0.42,1.03) | 0.068 | 0.024 |
| **CLL (C91)** | 301,166 (308) | 2,137,248 | 0.82 (0.70,0.97) | 0.024 |  | 85,205 (78) | 603,491 | 1.02 (0.68,1.54) | 0.924 | 0.986 |

Adjusted for age, sex (female, male), ethnic (White, Asian, African, mixed background, unknown), education (no degree, degree, unknown), Townsend deprivation index, standing height, BMI, smoking status (never, previous, current, unknown), alcohol use (never, previous, current, unknown), physical activity (<600 MET/week, 600-3,000 MET/week, ≥3,000 MET/week), family cancer (no, yes), and assessment centre. Additionally adjusted for menopausal (no, yes, not sure, unknown), oral contraceptive use (never, ever, unknown), hormone replacement therapy (never, ever, unknown) for female. CNS, central nervous system; CLL, chronic lymphocytic leukemia.

**Table S7.** Sensitivity analysis of the observational association between the CRP and cancer outcomes by additionally adjusting for cardiovascular and diabetes diseases^*^

|  | **≤ 3mg/L** | | | |  | **>3 mg/L** | | | | |
| --- | --- | --- | --- | --- | --- | --- | --- | --- | --- | --- |
|  | **No**  **(incident cases)** | **Person years** | **HR (95%CI)** | ***P*** |  | **No**  **(incident cases)** | **Person years** | **HR (95%CI)** | ***P*** | ***P* _heterogeneity_** |
| **Overall cancer** | 327,031 (26,173) | 2,237,479 | 1.04 (1.03,1.06) | <0.001 |  | 93,933 (8,806) | 635,846 | 1.01 (1.01,1.01) | <0.001 | <0.001 |
| **Head & neck (C00-14)** | 301,209 (351) | 2,137,461 | 1.08 (0.93,1.25) | 0.320 |  | 85,283 (156) | 603,797 | 1.02 (0.99,1.05) | 0.186 | 0.482 |
| **Esophagus (C15)** | 301,201 (344) | 2,137,476 | 1.14 (0.98,1.32) | 0.083 |  | 85,273 (146) | 603,701 | 1.04 (1.01,1.07) | 0.017 | 0.221 |
| **Stomach (C16)** | 301,087 (229) | 2,136,975 | 1.05 (0.87,1.26) | 0.618 |  | 85,232 (106) | 603,559 | 1.03 (0.99,1.07) | 0.150 | 0.839 |
| **Colorectal (C18-20)** | 303,003 (2,145) | 2,144,527 | 1.11 (1.04,1.18) | 0.001 |  | 85,917 (791) | 605,899 | 1.02 (1.00,1.03) | 0.027 | 0.005 |
| **Liver (C22)** | 301,037 (179) | 2,136,852 | 1.20 (0.98,1.46) | 0.083 |  | 85,230 (103) | 603,594 | 1.00 (0.96,1.05) | 0.829 | 0.097 |
| **Gallbladder (C23-24)** | 300,952 (94) | 2,136,473 | 0.87 (0.65,1.17) | 0.369 |  | 85,172 (45) | 603,354 | 0.91 (0.82,1.00) | 0.039 | 0.820 |
| **Pancreas (C25)** | 301,268 (410) | 2,137,813 | 1.13 (0.99,1.30) | 0.069 |  | 85,286 (159) | 603,798 | 0.99 (0.95,1.02) | 0.495 | 0.054 |
| **Lung (C33-34)** | 301,995 (1,137) | 2,140,855 | 1.26 (1.16,1.36) | <0.001 |  | 85,882 (755) | 606,121 | 1.03 (1.02,1.05) | <0.001 | <0.001 |
| **Melanoma (C43)** | 301,966 (1,111) | 2,140,358 | 0.98 (0.90,1.07) | 0.692 |  | 85,437 (310) | 604,460 | 1.01 (0.99,1.04) | 0.262 | 0.504 |
| **Non-melanotic skin (C44)** | 309,450 (8,597) | 2,170,575 | 1.01 (0.97,1.04) | 0.732 |  | 87,430 (2,305) | 612,401 | 1.01 (1.00,1.02) | 0.143 | 0.954 |
| **Breast (C50)** | 160,803 (3,644) | 1,130,896 | 1.07 (1.03,1.13) | 0.003 |  | 51,446 (1,371) | 361,233 | 0.99 (0.98, 1.00) | 0.132 | 0.001 |
| **Uterus (C54-55)** | 157,651 (491) | 1,119,136 | 1.13 (1.00,1.28) | 0.050 |  | 50,382 (306) | 357,326 | 1.01 (0.99,1.04) | 0.309 | 0.082 |
| **Ovary (C56)** | 157,548 (388) | 1,118,715 | 1.12 (0.97,1.29) | 0.129 |  | 50,223 (147) | 356,717 | 1.00 (0.96,1.04) | 0.998 | 0.141 |
| **Prostate (C61)** | 148,034 (4,337) | 1,035,523 | 0.99 (0.95,1.03) | 0.641 |  | 36,092 (1,043) | 250,894 | 1.00 (0.99,1.01) | 0.818 | 0.703 |
| **Kidney (C64)** | 301,320 (463) | 2,137,990 | 1.13 (0.99,1.28) | 0.068 |  | 85,362 (235) | 604,047 | 1.05 (1.03,1.07) | <0.001 | 0.283 |
| **Bladder (C67)** | 301,270 (412) | 2,137,591 | 1.05 (0.91,1.20) | 0.526 |  | 85,281 (154) | 603,785 | 1.01 (0.98,1.05) | 0.407 | 0.669 |
| **CNS (C70-72)** | 301,185 (327) | 2,137,370 | 1.14 (0.98,1.33) | 0.093 |  | 85,232 (105) | 603,587 | 0.96 (0.92,1.01) | 0.118 | 0.038 |
| **Thyroid (C73)** | 301,031 (173) | 2,136,726 | 1.06 (0.86,1.31) | 0.582 |  | 85,179 (52) | 603,385 | 1.01 (0.96,1.07) | 0.616 | 0.686 |
| **Non-Hodgkin lymphoma (C82-85,96)** | 301,634 (776) | 2,139,208 | 1.08 (0.98,1.20) | 0.120 |  | 85,447 (320) | 604,276 | 1.02 (1.00,1.05) | 0.042 | 0.274 |
| **Multiple myeloma (C90)** | 301,172 (315) | 2,137,379 | 0.90 (0.76,1.05) | 0.183 |  | 85,211 (84) | 603,538 | 0.96 (0.91,1.02) | 0.183 | 0.398 |
| **CLL (C91)** | 301,166 (308) | 2,137,248 | 0.83 (0.70,0.99) | 0.035 |  | 85,205 (78) | 603,491 | 1.00 (0.95,1.05) | 0.949 | 0.044 |

^*^Hazard ratios for cancer outcomes per 1 mg/L higher CRP

Adjusted for age, sex (female, male), ethnic (White, Asian, African, mixed background, unknown), education (no degree, degree, unknown), Townsend deprivation index, standing height, BMI, smoking status (never, previous, current, unknown), alcohol use (never, previous, current, unknown), physical activity (<600 MET/week, 600-3,000 MET/week, ≥3,000 MET/week), family cancer (no, yes), assessment centre, cardiovascular, and diabetes diseases. Additionally adjusted for menopausal (no, yes, not sure, unknown), oral contraceptive use (never, ever, unknown), hormone replacement therapy (never, ever, unknown) for female. CNS, central nervous system; CLL, chronic lymphocytic leukemia.

**Table S8.** Sensitivity analysis of the observational association between the CRP and cancer outcomes by additionally adjusting for drugs^*^

|  | **≤ 3mg/L** | | | |  | **>3 mg/L** | | | | |
| --- | --- | --- | --- | --- | --- | --- | --- | --- | --- | --- |
|  | **No**  **(incident cases)** | **Person years** | **HR (95%CI)** | ***P*** |  | **No**  **(incident cases)** | **Person years** | **HR (95%CI)** | ***P*** | ***P* _heterogeneity_** |
| **Overall cancer** | 327,031 (26,173) | 2,237,479 | 1.04 (1.03,1.06) | <0.001 |  | 93,933 (8,806) | 635,846 | 1.01 (1.01,1.01) | <0.001 | <0.001 |
| **Head & neck (C00-14)** | 301,209 (351) | 2,137,461 | 1.08 (0.93,1.25) | 0.324 |  | 85,283 (156) | 603,797 | 1.02 (0.99,1.05) | 0.179 | 0.489 |
| **Esophagus (C15)** | 301,201 (344) | 2,137,476 | 1.14 (0.98,1.32) | 0.084 |  | 85,273 (146) | 603,701 | 1.04 (1.01,1.07) | 0.014 | 0.229 |
| **Stomach (C16)** | 301,087 (229) | 2,136,975 | 1.06 (0.88,1.27) | 0.560 |  | 85,232 (106) | 603,559 | 1.03 (0.99,1.07) | 0.155 | 0.773 |
| **Colorectal (C18-20)** | 303,003 (2,145) | 2,144,527 | 1.11 (1.04,1.18) | 0.001 |  | 85,917 (791) | 605,899 | 1.02 (1.00,1.03) | 0.026 | 0.006 |
| **Liver (C22)** | 301,037 (179) | 2,136,852 | 1.21 (0.99,1.48) | 0.064 |  | 85,230 (103) | 603,594 | 1.01 (0.97,1.05) | 0.795 | 0.078 |
| **Gallbladder (C23-24)** | 300,952 (94) | 2,136,473 | 0.87 (0.65,1.17) | 0.364 |  | 85,172 (45) | 603,354 | 0.9 (0.82,0.99) | 0.034 | 0.828 |
| **Pancreas (C25)** | 301,268 (410) | 2,137,813 | 1.13 (0.99,1.30) | 0.068 |  | 85,286 (159) | 603,798 | 0.99 (0.95,1.02) | 0.507 | 0.053 |
| **Lung (C33-34)** | 301,995 (1,137) | 2,140,855 | 1.27 (1.17,1.37) | <0.001 |  | 85,882 (755) | 606,121 | 1.03 (1.02,1.05) | <0.001 | <0.001 |
| **Melanoma (C43)** | 301,966 (1,111) | 2,140,358 | 0.98 (0.90,1.07) | 0.662 |  | 85,437 (310) | 604,460 | 1.01 (0.99,1.04) | 0.241 | 0.471 |
| **Non-melanotic skin (C44)** | 309,450 (8,597) | 2,170,575 | 1.01 (0.98,1.04) | 0.689 |  | 87,430 (2,305) | 612,401 | 1.01 (1.00,1.02) | 0.127 | 0.985 |
| **Breast (C50)** | 160,803 (3,644) | 1,130,896 | 1.07 (1.02,1.13) | 0.003 |  | 51,446 (1,371) | 361,233 | 0.99 (0.98,1.00) | 0.137 | 0.001 |
| **Uterus (C54-55)** | 157,651 (491) | 1,119,136 | 1.14 (1.00,1.29) | 0.046 |  | 50,382 (306) | 357,326 | 1.01 (0.99,1.04) | 0.318 | 0.075 |
| **Ovary (C56)** | 157,548 (388) | 1,118,715 | 1.12 (0.97,1.29) | 0.130 |  | 50,223 (147) | 356,717 | 1.00 (0.96,1.04) | 0.998 | 0.143 |
| **Prostate (C61)** | 148,034 (4,337) | 1,035,523 | 0.99 (0.94,1.03) | 0.555 |  | 36,092 (1,043) | 250,894 | 1.00 (0.99,1.01) | 0.797 | 0.621 |
| **Kidney (C64)** | 301,320 (463) | 2,137,990 | 1.14 (1.00,1.29) | 0.048 |  | 85,362 (235) | 604,047 | 1.05 (1.03,1.07) | <0.001 | 0.222 |
| **Bladder (C67)** | 301,270 (412) | 2,137,591 | 1.05 (0.92,1.21) | 0.469 |  | 85,281 (154) | 603,785 | 1.01 (0.98,1.05) | 0.390 | 0.611 |
| **CNS (C70-72)** | 301,185 (327) | 2,137,370 | 1.13 (0.97,1.32) | 0.110 |  | 85,232 (105) | 603,587 | 0.96 (0.92,1.01) | 0.113 | 0.046 |
| **Thyroid (C73)** | 301,031 (173) | 2,136,726 | 1.07 (0.86,1.32) | 0.543 |  | 85,179 (52) | 603,385 | 1.01 (0.96,1.07) | 0.609 | 0.648 |
| **Non-Hodgkin lymphoma (C82-85,96)** | 301,634 (776) | 2,139,208 | 1.08 (0.98,1.20) | 0.118 |  | 85,447 (320) | 604,276 | 1.02 (1.00,1.05) | 0.040 | 0.271 |
| **Multiple myeloma (C90)** | 301,172 (315) | 2,137,379 | 0.90 (0.76,1.06) | 0.192 |  | 85,211 (84) | 603,538 | 0.96 (0.91,1.02) | 0.181 | 0.412 |
| **CLL (C91)** | 301,166 (308) | 2,137,248 | 0.83 (0.70,0.98) | 0.028 |  | 85,205 (78) | 603,491 | 1.00 (0.95,1.05) | 0.922 | 0.036 |

^*^Hazard ratios for cancer outcomes per 1 mg/L higher CRP

Adjusted for age, sex (female, male), ethnic (White, Asian, African, mixed background, unknown), education (no degree, degree, unknown), Townsend deprivation index, standing height, BMI, smoking status (never, previous, current, unknown), alcohol use (never, previous, current, unknown), physical activity (<600 MET/week, 600-3,000 MET/week, ≥3,000 MET/week), family cancer (no, yes), assessment centre, cardiovascular, diabetes diseases, regular use of aspirin and ibuprofen. Additionally adjusted for menopausal (no, yes, not sure, unknown), oral contraceptive use (never, ever, unknown), hormone replacement therapy (never, ever, unknown) for female. CNS, central nervous system; CLL, chronic lymphocytic leukemia.

**Table S9.** Sensitivity analysis of the observational association between the CRP and cancer outcomes by excluding of patients diagnosed in the first two follow-up^*^

|  | **≤ 3mg/L** | | | |  | **>3 mg/L** | | | | |
| --- | --- | --- | --- | --- | --- | --- | --- | --- | --- | --- |
|  | **No**  **(incident cases)** | **Person years** | **HR (95%CI)** | ***P*** |  | **No**  **(incident cases)** | **Person years** | **HR (95%CI)** | ***P*** | ***P* _heterogeneity_** |
| **Overall cancer** | 320,949 (20,091) | 320,949 | 1.04 (1.02,1.06) | 0.001 |  | 91,579 (6,452) | 633,509 | 1.01 (1.00,1.01) | 0.006 | 0.008 |
| **Head & neck (C00-14)** | 301,130 (272) | 301,130 | 1.10 (0.93,1.30) | 0.276 |  | 122 (85,127) | 603,755 | 1.02 (0.99,1.06) | 0.215 | 0.416 |
| **Esophagus (C15)** | 301,134 (277) | 301,134 | 1.14 (0.97,1.35) | 0.105 |  | 108 (85,127) | 603,658 | 1.04 (1.00,1.07) | 0.050 | 0.242 |
| **Stomach (C16)** | 301,043 (185) | 301,043 | 0.95 (0.77,1.17) | 0.619 |  | 74 (85,126) | 603,526 | 1.01 (0.96,1.06) | 0.733 | 0.575 |
| **Colorectal (C18-20)** | 302,545 (1,687) | 302,545 | 1.07 (1.00,1.15) | 0.048 |  | 543 (85,126) | 605,658 | 1.02 (1.00,1.03) | 0.070 | 0.139 |
| **Liver (C22)** | 301,008 (150) | 301,008 | 1.19 (0.96,1.49) | 0.119 |  | 77 (85,127) | 603,568 | 1.00 (0.96,1.05) | 0.840 | 0.138 |
| **Gallbladder (C23-24)** | 300,938 (80) | 300,938 | 0.80 (0.58,1.11) | 0.178 |  | 33 (85,127) | 603,341 | 0.88 (0.78,0.99) | 0.041 | 0.582 |
| **Pancreas (C25)** | 301,200 (342) | 301,200 | 1.10 (0.95,1.28) | 0.219 |  | 119 (85,127) | 603,756 | 0.96 (0.92,1.01) | 0.088 | 0.094 |
| **Lung (C33-34)** | 301,795 (937) | 301,795 | 1.24 (1.14,1.35) | <0.001 |  | 577 (85,127) | 605,941 | 1.03 (1.01,1.04) | 0.001 | <0.001 |
| **Melanoma (C43)** | 301,690 (835) | 301,690 | 0.92 (0.83,1.02) | 0.100 |  | 239 (85,127) | 604,379 | 1.02 (0.99,1.04) | 0.235 | 0.059 |
| **Non-melanotic skin (C44)** | 307,580 (6,727) | 307,580 | 1.02 (0.98,1.05) | 0.319 |  | 1,813 (85,125) | 611,914 | 1.01 (1.00,1.02) | 0.187 | 0.543 |
| **Breast (C50)** | 159,890 (2,731) | 159,890 | 1.07 (1.01,1.13) | 0.018 |  | 1,000 (50,075) | 360,866 | 0.99 (0.98,1.00) | 0.167 | 0.008 |
| **Uterus (C54-55)** | 157,532 (372) | 157,532 | 1.13 (0.98,1.30) | 0.097 |  | 226 (50,076) | 357,248 | 1.02 (0.99,1.05) | 0.161 | 0.169 |
| **Ovary (C56)** | 157,454 (294) | 157,454 | 1.07 (0.90,1.26) | 0.439 |  | 100 (50,076) | 356,672 | 0.99 (0.95,1.04) | 0.767 | 0.409 |
| **Prostate (C61)** | 147,039 (3,342) | 147,039 | 1.00 (0.95,1.05) | 0.926 |  | 766 (35,049) | 250,606 | 1.00 (0.98,1.01) | 0.644 | 0.823 |
| **Kidney (C64)** | 301,242 (385) | 301,242 | 1.16 (1.01,1.33) | 0.038 |  | 169 (85,127) | 603,976 | 1.04 (1.01,1.07) | 0.013 | 0.123 |
| **Bladder (C67)** | 301,166 (308) | 301,166 | 1.06 (0.91,1.24) | 0.468 |  | 112 (85,127) | 603,742 | 1.02 (0.98,1.06) | 0.252 | 0.653 |
| **CNS (C70-72)** | 301,108 (250) | 301,108 | 1.06 (0.88,1.26) | 0.555 |  | 82 (85,127) | 603,567 | 0.98 (0.93,1.03) | 0.432 | 0.434 |
| **Thyroid (C73)** | 300,984 (126) | 300,984 | 0.97 (0.75,1.26) | 0.839 |  | 40 (85,127) | 603,372 | 0.97 (0.90,1.05) | 0.472 | 0.991 |
| **Non-Hodgkin lymphoma**  **(C82-85,96)** | 301,483 (625) | 301,483 | 1.03 (0.92,1.16) | 0.578 |  | 216 (85,127) | 604,173 | 1.01 (0.98,1.04) | 0.529 | 0.696 |
| **Multiple myeloma (C90)** | 301,116 (259) | 301,116 | 0.84 (0.70,1.01) | 0.068 |  | 67 (85,127) | 603,520 | 0.97 (0.91,1.03) | 0.302 | 0.156 |
| **CLL (C91)** | 301,084 (226) | 301,084 | 0.76 (0.62,0.93) | 0.008 |  | 62 (85,127) | 603,471 | 0.98 (0.92,1.04) | 0.496 | 0.018 |

^*^Hazard ratios for cancer outcomes per 1 mg/L higher CRP

Adjusted for age, sex (female, male), ethnic (White, Asian, African, mixed background, unknown), education (no degree, degree, unknown), Townsend deprivation index, standing height, BMI, smoking status (never, previous, current, unknown), alcohol use (never, previous, current, unknown), physical activity (<600 MET/week, 600-3,000 MET/week, ≥3,000 MET/week), family cancer (no, yes), and assessment centre. Additionally adjusted for menopausal (no, yes, not sure, unknown), oral contraceptive use (never, ever, unknown), hormone replacement therapy (never, ever, unknown) for female. CNS, central nervous system; CLL, chronic lymphocytic leukemia

**Table S10.** Sensitivity analysis of the observational association between the CRP and cancer outcomes by only including of patients diagnosed in the first two follow-up^*^

|  | **≤ 3mg/L** | | | |  | **>3 mg/L** | | | | |
| --- | --- | --- | --- | --- | --- | --- | --- | --- | --- | --- |
|  | **No (incident cases)** | **Person years** | **HR (95%CI)** | ***P*** |  | **No (incident cases)** | **Person years** | **HR (95%CI)** | ***P*** | ***P* _heterogeneity_** |
| **Overall cancer** | 306,940 (6,082) | 2,142,372 | 1.07 (1.04,1.11) | <0.001 |  | 87,481 (2,354) | 605,545 | 1.02 (1.01,1.03) | <0.001 | 0.006 |
| **Head & neck (C00-14)** | 300,937 (79) | 2,136,146 | 1.01 (0.74,1.39) | 0.929 |  | 85,161 (34) | 603,229 | 1.02 (0.95,1.09) | 0.578 | 0.979 |
| **Esophagus (C15)** | 300,924 (67) | 2,136,128 | 1.13 (0.81,1.57) | 0.479 |  | 85,165 (38) | 603,230 | 1.04 (0.98,1.10) | 0.160 | 0.643 |
| **Stomach (C16)** | 300,902 (44) | 2,136,111 | 1.56 (1.05,2.31) | 0.026 |  | 85,158 (32) | 603,214 | 1.06 (1.00,1.13) | 0.047 | 0.057 |
| **Colorectal (C18-20)** | 301,316 (458) | 2,136,564 | 1.25 (1.10,1.42) | <0.001 |  | 85,374 (248) | 603,421 | 1.02 (0.99,1.04) | 0.228 | 0.001 |
| **Liver (C22)** | 301,037 (179) | 2,136,852 | 1.20 (0.98,1.47) | 0.080 |  | 85,230 (103) | 603,594 | 1.01 (0.97,1.05) | 0.788 | 0.096 |
| **Gallbladder (C23-24)** | 300,872 (14) | 2,136,080 | 1.34 (0.65,2.75) | 0.423 |  | 85,139 (12) | 603,200 | 0.96 (0.83,1.11) | 0.556 | 0.367 |
| **Pancreas (C25)** | 300,926 (68) | 2,136,142 | 1.33 (0.96,1.83) | 0.087 |  | 85,167 (40) | 603,228 | 1.05 (0.99,1.11) | 0.114 | 0.157 |
| **Lung (C33-34)** | 301,058 (200) | 2,136,299 | 1.35 (1.12,1.63) | 0.001 |  | 85,305 (178) | 603,367 | 1.06 (1.03,1.08) | <0.001 | 0.009 |
| **Melanoma (C43)** | 301,131 (276) | 2,136,315 | 1.18 (1.00,1.40) | 0.048 |  | 85,198 (71) | 603,268 | 1.01 (0.96,1.06) | 0.780 | 0.070 |
| **Non-melanotic skin (C44)** | 302,723 (1,870) | 2,137,920 | 0.96 (0.90,1.03) | 0.258 |  | 85,617 (492) | 603,660 | 1.01 (0.99,1.03) | 0.436 | 0.195 |
| **Breast (C50)** | 158,072 (913) | 1,118,187 | 1.09 (1.00,1.20) | 0.059 |  | 50,446 (371) | 356,570 | 0.99 (0.97,1.02) | 0.542 | 0.048 |
| **Uterus (C54-55)** | 157,279 (119) | 1,117,430 | 1.14 (0.89,1.47) | 0.307 |  | 50,156 (80) | 356,288 | 0.99 (0.94,1.04) | 0.683 | 0.280 |
| **Ovary (C56)** | 157,254 (94) | 1,117,398 | 1.29 (0.97,1.71) | 0.079 |  | 50,123 (47) | 356,255 | 1.01 (0.95,1.08) | 0.648 | 0.105 |
| **Prostate (C61)** | 144,692 (995) | 1,019,811 | 0.95 (0.87,1.04) | 0.256 |  | 35,326 (277) | 247,248 | 1.00 (0.98,1.03) | 0.782 | 0.243 |
| **Kidney (C64)** | 300,935 (78) | 2,136,149 | 0.98 (0.71,1.35) | 0.895 |  | 85,193 (66) | 603,257 | 1.08 (1.03,1.12) | <0.001 | 0.567 |
| **Bladder (C67)** | 300,962 (104) | 2,136,181 | 1.01 (0.77,1.33) | 0.945 |  | 85,169 (42) | 603,230 | 0.99 (0.93,1.06) | 0.803 | 0.900 |
| **CNS (C70-72)** | 300,935 (77) | 2,136,146 | 1.45 (1.07,1.96) | 0.017 |  | 85,150 (23) | 603,207 | 0.86 (0.74,1.01) | 0.073 | 0.003 |
| **Thyroid (C73)** | 300,905 (47) | 2,136,116 | 1.34 (0.90,1.98) | 0.149 |  | 85,139 (12) | 603,200 | 1.11 (1.01,1.21) | 0.028 | 0.360 |
| **Non-Hodgkin lymphoma**  **(C82-85,96)** | 301,009 (151) | 2,136,234 | 1.30 (1.04,1.62) | 0.019 |  | 85,231 (104) | 603,290 | 1.05 (1.01,1.09) | 0.008 | 0.057 |
| **Multiple myeloma (C90)** | 300,913 (56) | 2,136,126 | 1.15 (0.79,1.66) | 0.468 |  | 85,144 (17) | 603,204 | 0.94 (0.82,1.07) | 0.350 | 0.316 |
| **CLL (C91)** | 300,940 (82) | 2,136,148 | 1.04 (0.76,1.43) | 0.785 |  | 85,143 (16) | 603,206 | 1.04 (0.95,1.14) | 0.381 | 0.986 |

^*^Hazard ratios for cancer outcomes per 1 mg/L higher CRP

Adjusted for age, sex (female, male), ethnic (White, Asian, African, mixed background, unknown), education (no degree, degree, unknown), Townsend deprivation index, standing height, BMI, smoking status (never, previous, current, unknown), alcohol use (never, previous, current, unknown), physical activity (<600 MET/week, 600-3,000 MET/week, ≥3,000 MET/week), family cancer (no, yes), and assessment centre. Additionally adjusted for menopausal (no, yes, not sure, unknown), oral contraceptive use (never, ever, unknown), hormone replacement therapy (never, ever, unknown) for female. CNS, central nervous system; CLL, chronic lymphocytic leukemia

**Table S11.** Sensitivity analysis of the observational association between the CRP and cancer outcomes by excluding of participants with CRP > 10 mg/L^*^

|  | **≤ 3mg/L** | | | |  |  | **> 3 mg/L** | | | |
| --- | --- | --- | --- | --- | --- | --- | --- | --- | --- | --- |
|  | **No (incident cases)** | **Person years** | **HR (95%CI)** | ***P* value** |  | **No (incident cases)** | **Person**  **years** | **HR (95%CI)** | ***P* value** | ***P* _heterogeneity_** |
| **Overall cancer** | 327,031 (26,173) | 2,237,479 | 1.04 (1.03,1.06) | <0.001 |  | 77,036 (7,100) | 522,628 | 1.02 (1.01,1.03) | 0.007 | 0.035 |
| **Head & neck (C00-14)** | 301,209 (351) | 2,137,461 | 1.08 (0.93,1.25) | 0.318 |  | 70,056 (120) | 496,561 | 1.03 (0.93,1.15) | 0.546 | 0.643 |
| **Esophagus (C15)** | 301,201 (344) | 2,137,476 | 1.14 (0.99,1.32) | 0.078 |  | 70,049 (113) | 496,483 | 1.05 (0.94,1.17) | 0.410 | 0.353 |
| **Stomach (C16)** | 301,087 (229) | 2,136,975 | 1.05 (0.87,1.26) | 0.614 |  | 70,022 (86) | 496,413 | 1.22 (1.09,1.37) | 0.001 | 0.169 |
| **Colorectal (C18-20)** | 303,003 (2,145) | 2,144,527 | 1.11 (1.04,1.18) | 0.001 |  | 70,566 (631) | 498,249 | 1.01 (0.97,1.06) | 0.546 | 0.022 |
| **Liver (C22)** | 301,037 (179) | 2,136,852 | 1.20 (0.98,1.47) | 0.080 |  | 70,018 (82) | 496,403 | 1.00 (0.88,1.14) | 0.961 | 0.149 |
| **Gallbladder (C23-24)** | 300,952 (94) | 2,136,473 | 0.87 (0.65,1.17) | 0.351 |  | 69,978 (42) | 496,248 | 0.89 (0.73,1.09) | 0.257 | 0.884 |
| **Pancreas (C25)** | 301,268 (410) | 2,137,813 | 1.14 (0.99,1.30) | 0.066 |  | 70,072 (136) | 496,629 | 1.00 (0.90,1.11) | 0.985 | 0.137 |
| **Lung (C33-34)** | 301,995 (1,137) | 2,140,855 | 1.26 (1.16,1.36) | <0.001 |  | 70,506 (570) | 498,313 | 1.10 (1.05,1.15) | <0.001 | 0.004 |
| **Melanoma (C43)** | 301,966 (1,111) | 2,140,358 | 0.98 (0.90,1.07) | 0.655 |  | 70,185 (249) | 497,104 | 1.04 (0.97,1.12) | 0.284 | 0.302 |
| **Non-melanotic skin (C44)** | 309,450 (8,597) | 2,170,575 | 1.01 (0.97,1.04) | 0.729 |  | 71,800 (1,866) | 503,595 | 1.00 (0.98,1.03) | 0.757 | 0.957 |
| **Breast (C50)** | 160,803 (3,644) | 1,130,896 | 1.07 (1.02,1.13) | 0.003 |  | 42,183 (1,141) | 296,255 | 0.96 (0.93,1.00) | 0.044 | <0.001 |
| **Uterus (C54-55)** | 157,651 (491) | 1,119,136 | 1.13 (1.00,1.28) | 0.050 |  | 41,285 (242) | 292,924 | 1.05 (0.98,1.13) | 0.175 | 0.307 |
| **Ovary (C56)** | 157,548 (388) | 1,118,715 | 1.12 (0.97,1.29) | 0.126 |  | 41,164 (121) | 292,461 | 0.98 (0.88,1.10) | 0.784 | 0.166 |
| **Prostate (C61)** | 148,034 (4,337) | 1,035,523 | 0.99 (0.95,1.03) | 0.649 |  | 29,752 (861) | 207,284 | 1.02 (0.98,1.06) | 0.289 | 0.292 |
| **Kidney (C64)** | 301,320 (463) | 2,137,990 | 1.13 (0.99,1.28) | 0.066 |  | 70,113 (177) | 496,744 | 1.14 (1.05,1.24) | 0.002 | 0.875 |
| **Bladder (C67)** | 301,270 (412) | 2,137,591 | 1.05 (0.91,1.20) | 0.524 |  | 70,060 (124) | 496,561 | 1.00 (0.90,1.12) | 0.940 | 0.647 |
| **CNS (C70-72)** | 301,185 (327) | 2,137,370 | 1.14 (0.98,1.33) | 0.092 |  | 70,028 (92) | 496,430 | 0.94 (0.82,1.07) | 0.322 | 0.054 |
| **Thyroid (C73)** | 301,031 (173) | 2,136,726 | 1.07 (0.86,1.32) | 0.553 |  | 69,978 (42) | 496,251 | 1.17 (0.99,1.38) | 0.067 | 0.508 |
| **Non-Hodgkin lymphoma**  **(C82-85,96)** | 301,634 (776) | 2,139,208 | 1.08 (0.98,1.20) | 0.123 |  | 70,191 (255) | 496,978 | 1.07 (0.99,1.14) | 0.082 | 0.799 |
| **Multiple myeloma (C90)** | 301,172 (315) | 2,137,379 | 0.89 (0.76,1.05) | 0.182 |  | 70,009 (73) | 496,389 | 0.88 (0.76,1.03) | 0.108 | 0.901 |
| **CLL (C91)** | 301,166 (308) | 2,137,248 | 0.83 (0.70,0.98) | 0.033 |  | 70,002 (66) | 496,345 | 1.07 (0.93,1.22) | 0.367 | 0.026 |

^*^Hazard ratios for cancer outcomes per 1 mg/L higher CRP

Adjusted for age, sex (female, male), ethnic (White, Asian, African, mixed background, unknown), education (no degree, degree, unknown), Townsend deprivation index, standing height, BMI, smoking status (never, previous, current, unknown), alcohol use (never, previous, current, unknown), physical activity (<600 MET/week, 600-3,000 MET/week, ≥3,000 MET/week), family cancer (no, yes), and assessment centre. Additionally adjusted for menopausal (no, yes, not sure, unknown), oral contraceptive use (never, ever, unknown), hormone replacement therapy (never, ever, unknown) for female. CNS, central nervous system; CLL, chronic lymphocytic leukemia

**Table S12.** Subgroup analysis of association between CRP and cancer risk

|  | **Low CRP** | **Average CRP** | **High CRP** | ***P* _interaction_** |
| --- | --- | --- | --- | --- |
|  | **(<1 mg/L)** | **(1 to 3 mg/L)** | **(>3 mg/L)** |  |
| **Overall cancer** |  |  |  |  |
| Age |  |  |  | <0.001 |
| ≤ 65 years | Reference | 1.02 (0.97,1.08) | 1.11 (1.05,1.18) |  |
| > 65 years | Reference | 1.12 (1.09,1.16) | 1.25 (1.21,1.30) |  |
| Sex |  |  |  |  |
| Female | Reference | 1.07 (1.03,1.11) | 1.16 (1.11,1.21) | 0.851 |
| Male | Reference | 1.03 (1.00,1.07) | 1.13 (1.08,1.18) |  |
| Smoking status |  |  |  |  |
| Never | Reference | 1.03 (1.00,1.07) | 1.09 (1.04,1.14) | <0.001 |
| Previous | Reference | 1.03 (0.99,1.07) | 1.15 (1.09,1.20) |  |
| Current | Reference | 1.17 (1.08,1.28) | 1.38 (1.27,1.51) |  |
| **Head & neck (C00-14)** |  |  |  |  |
| Age |  |  |  |  |
| ≤ 65 years | Reference | 0.59 (0.34,1.01) | 1.06 (0.62,1.82) | 0.049 |
| > 65 years | Reference | 1.22 (0.97,1.55) | 1.62 (1.24,2.11) |  |
| Sex |  |  |  |  |
| Female | Reference | 1.24 (0.85,1.82) | 1.40 (0.89,2.18) | 0.365 |
| Male | Reference | 0.97 (0.74,1.26) | 1.46 (1.10,1.93) |  |
| Smoking status |  |  |  |  |
| Never | Reference | 1.08 (0.76,1.55) | 1.00 (0.62,1.59) | 0.091 |
| Previous | Reference | 0.76 (0.54,1.08) | 1.20 (0.82,1.75) |  |
| Current | Reference | 1.53 (0.97,2.43) | 2.27 (1.43,3.59) |  |
| **Esophagus (C15)** |  |  |  |  |
| Age |  |  |  |  |
| ≤ 65 years | Reference | 1.09 (0.73,1.63) | 1.41 (0.91,2.18) | 0.380 |
| > 65 years | Reference | 1.34 (1.03,1.75) | 1.38 (1.01,1.88) |  |
| Sex |  |  |  |  |
| Female | Reference | 1.12 (0.71,1.75) | 1.31 (0.78,2.20) | 0.129 |
| Male | Reference | 1.24 (0.96,1.60) | 1.36 (1.02,1.82) |  |
| Smoking status |  |  |  |  |
| Never | Reference | 1.07 (0.73,1.57) | 1.22 (0.77,1.93) | 0.520 |
| Previous | Reference | 1.14 (0.84,1.55) | 1.14 (0.80,1.64) |  |
| Current | Reference | 1.84 (0.99,3.41) | 2.21 (1.18,4.13) |  |
| **Stomach (C16)** |  |  |  |  |
| Age |  |  |  |  |
| ≤ 65 years | Reference | 0.79 (0.50,1.26) | 0.95 (0.57,1.59) | 0.093 |
| > 65 years | Reference | 1.39 (1.00,1.93) | 1.85 (1.28,2.67) |  |
| Sex |  |  |  |  |
| Female | Reference | 0.67 (0.40,1.11) | 1.03 (0.59,1.77) | 0.067 |
| Male | Reference | 1.32 (0.96,1.83) | 1.57 (1.09,2.25) |  |
| Smoking status |  |  |  |  |
| Never | Reference | 0.81 (0.53,1.24) | 1.45 (0.90,2.31) | 0.128 |
| Previous | Reference | 1.41 (0.92,2.16) | 1.52 (0.94,2.45) |  |
| Current | Reference | 1.13 (0.57,2.27) | 1.33 (0.65,2.72) |  |
| **Colorectal (C18-20)** |  |  |  |  |
| Age |  |  |  |  |
| ≤ 65 years | Reference | 0.99 (0.84,1.17) | 1.15 (0.95,1.39) | 0.061 |
| > 65 years | Reference | 1.24 (1.12,1.37) | 1.42 (1.26,1.61) |  |
| Sex |  |  |  |  |
| Female | Reference | 1.13 (0.98,1.29) | 1.18 (1.00,1.39) | 0.035 |
| Male | Reference | 1.10 (0.98,1.23) | 1.34 (1.18,1.53) |  |
| Smoking status |  |  |  |  |
| Never | Reference | 1.18 (1.04,1.34) | 1.34 (1.14,1.56) | 0.444 |
| Previous | Reference | 1.04 (0.91,1.19) | 1.25 (1.07,1.45) |  |
| Current | Reference | 0.98 (0.73,1.32) | 1.01 (0.74,1.38) |  |
| **Liver (C22)** |  |  |  |  |
| Age |  |  |  |  |
| ≤ 65 years | Reference | 1.53 (0.79,2.94) | 2.62 (1.35,5.11) | 0.253 |
| > 65 years | Reference | 1.10 (0.77,1.55) | 1.66 (1.13,2.43) |  |
| Sex |  |  |  |  |
| Female | Reference | 1.42 (0.83,2.44) | 2.24 (1.25,4.00) | 0.767 |
| Male | Reference | 0.99 (0.68,1.42) | 1.57 (1.06,2.32) |  |
| Smoking status |  |  |  |  |
| Never | Reference | 1.13 (0.70,1.82) | 1.41 (0.80,2.48) | 0.560 |
| Previous | Reference | 1.02 (0.66,1.60) | 1.95 (1.24,3.07) |  |
| Current | Reference | 1.24 (0.54,2.87) | 1.45 (0.61,3.44) |  |
| **Gallbladder (C23-24)** |  |  |  |  |
| Age |  |  |  |  |
| ≤ 65 years | Reference | 0.82 (0.36,1.83) | 0.96 (0.39,2.34) | 0.933 |
| > 65 years | Reference | 0.90 (0.55,1.46) | 1.22 (0.71,2.09) |  |
| Sex |  |  |  |  |
| Female | Reference | 0.75 (0.40,1.04) | 1.25 (0.65,2.40) | 0.707 |
| Male | Reference | 0.87 (0.50,1.53) | 0.89 (0.46,1.72) |  |
| Smoking status |  |  |  |  |
| Never | Reference | 1.06 (0.57,1.97) | 1.09 (0.52,2.29) | 0.499 |
| Previous | Reference | 0.51 (0.27,0.96) | 0.80 (0.41,1.55) |  |
| Current | Reference | 1.86 (0.68,5.11) | 2.57 (0.96,6.85) |  |
| **Pancreas (C25)** |  |  |  |  |
| Age |  |  |  |  |
| ≤ 65 years | Reference | 0.92 (0.63,1.34) | 1.09 (0.71,1.66) | 0.172 |
| > 65 years | Reference | 1.36 (1.07,1.73) | 1.34 (1.00,1.78) |  |
| Sex |  |  |  |  |
| Female | Reference | 1.33 (0.96,1.83) | 1.38 (0.96,1.99) | 0.371 |
| Male | Reference | 1.06 (0.81,1.38) | 1.05 (0.77,1.45) |  |
| Smoking status |  |  |  |  |
| Never | Reference | 1.39 (1.03,1.88) | 1.43 (0.99,2.06) | 0.133 |
| Previous | Reference | 0.86 (0.62,1.20) | 1.07 (0.74,1.55) |  |
| Current | Reference | 1.35 (0.80,2.29) | 1.02 (0.57,1.84) |  |
| **Lung (C33-34)** |  |  |  |  |
| Age |  |  |  |  |
| ≤ 65 years | Reference | 1.31 (1.04,1.65) | 1.87 (1.48,2.37) | 0.312 |
| > 65 years | Reference | 1.53 (1.32,1.77) | 2.45 (2.10,2.85) |  |
| Sex |  |  |  |  |
| Female | Reference | 1.14 (0.95,1.36) | 1.50 (1.24,1.82) | <0.001 |
| Male | Reference | 1.62 (1.36,1.93) | 2.65 (2.22,3.16) |  |
| Smoking status |  |  |  |  |
| Never | Reference | 0.86 (0.65,1.14) | 0.96 (0.68,1.37) | <0.001 |
| Previous | Reference | 1.45 (1.21,1.74) | 2.12 (1.75,2.58) |  |
| Current | Reference | 1.67 (1.34,2.09) | 2.74 (2.20,3.41) |  |
| **Melanoma (C43)** |  |  |  |  |
| Age |  |  |  |  |
| ≤ 65 years | Reference | 0.99 (0.75,1.30) | 1.07 (0.77,1.48) | 0.878 |
| > 65 years | Reference | 1.05 (0.91,1.20) | 1.07 (0.90,1.27) |  |
| Sex |  |  |  |  |
| Female | Reference | 1.09 (0.91,1.31) | 1.12 (0.90,1.40) | 0.701 |
| Male | Reference | 0.94 (0.79,1.11) | 0.99 (0.80,1.23) |  |
| Smoking status |  |  |  |  |
| Never | Reference | 1.08 (0.92,1.27) | 1.07 (0.86,1.32) | 0.103 |
| Previous | Reference | 0.99 (0.81,1.21) | 1.05 (0.82,1.33) |  |
| Current | Reference | 0.51 (0.29,0.90) | 0.82 (0.47,1.44) |  |
| **Non-melanotic skin (C44)** |  |  |  |  |
| Age |  |  |  |  |
| ≤ 65 years | Reference | 0.94 (0.86,1.03) | 0.95 (0.86,1.05) | 0.052 |
| > 65 years | Reference | 1.09 (1.03,1.15) | 1.10 (1.03,1.18) |  |
| Sex |  |  |  |  |
| Female | Reference | 1.01 (0.94,1.07) | 1.04 (0.96,1.13) | 0.638 |
| Male | Reference | 0.99 (0.93,1.05) | 0.96 (0.89,1.04) |  |
| Smoking status |  |  |  |  |
| Never | Reference | 0.96 (0.90,1.02) | 0.97 (0.89,1.05) | 0.062 |
| Previous | Reference | 1.03 (0.96,1.11) | 1.03 (0.94,1.12) |  |
| Current | Reference | 1.12 (0.95,1.33) | 1.11 (0.92,1.34) |  |
| **Breast (C50)** |  |  |  |  |
| Age |  |  |  |  |
| ≤ 65 years | Reference | 1.12 (0.93,1.36) | 1.33 (1.07,1.64) | 0.106 |
| > 65 years | Reference | 1.18 (1.10,1.27) | 1.20 (1.10,1.31) |  |
| Smoking status |  |  |  |  |
| Never | Reference | 1.17 (1.07,1.27) | 1.17 (1.05,1.30) | 0.128 |
| Previous | Reference | 1.12 (1.00,1.27) | 1.22 (1.06,1.40) |  |
| Current | Reference | 1.20 (0.93,1.54) | 1.37 (1.04,1.81) |  |
| **Uterus (C54-55)** |  |  |  |  |
| Age |  |  |  |  |
| ≤ 65 years | Reference | 1.07 (0.68,1.69) | 1.35 (0.83,2.21) | 0.851 |
| > 65 years | Reference | 1.04 (0.85,1.27) | 1.21 (0.96,1.52) |  |
| Smoking status |  |  |  |  |
| Never | Reference | 0.97 (0.77,1.21) | 1.04 (0.81,1.35) | 0.002 |
| Previous | Reference | 0.96 (0.68,1.36) | 1.31 (0.90,1.91) |  |
| Current | Reference | 5.18 (2.63,10.19) | 6.69 (3.44,13.0) |  |
| **Ovary (C56)** |  |  |  |  |
| Age |  |  |  |  |
| ≤ 65 years | Reference | 2.11 (1.28,3.47) | 1.96 (1.11,3.46) | 0.104 |
| > 65 years | Reference | 1.14 (0.90,1.44) | 1.29 (0.98,1.72) |  |
| Smoking status |  |  |  |  |
| Never | Reference | 1.38 (1.05,1.81) | 1.59 (1.14,2.21) | 0.317 |
| Previous | Reference | 1.08 (0.76,1.55) | 1.16 (0.76,1.78) |  |
| Current | Reference | 1.16 (0.53,2.50) | 1.02 (0.42,2.48) |  |
| **Prostate (C61)** |  |  |  |  |
| Age |  |  |  |  |
| ≤ 65 years | Reference | 1.02 (0.91,1.15) | 0.91 (0.79,1.05) | 0.064 |
| > 65 years | Reference | 1.11 (1.03,1.19) | 1.13 (1.03,1.24) |  |
| Smoking status |  |  |  |  |
| Never | Reference | 1.04 (0.95,1.13) | 0.92 (0.82,1.04) | 0.038 |
| Previous | Reference | 0.96 (0.87,1.05) | 0.96 (0.86,1.08) |  |
| Current | Reference | 0.96 (0.87,1.05) | 0.96 (0.86,1.08) |  |
| **Kidney (C64)** |  |  |  |  |
| Age |  |  |  |  |
| ≤ 65 years | Reference | 1.24 (0.84,1.83) | 1.34 (0.86,2.08) | 0.069 |
| > 65 years | Reference | 1.36 (1.09,1.69) | 1.90 (1.49,2.43) |  |
| Sex |  |  |  |  |
| Female | Reference | 1.63 (1.13,2.34) | 1.97 (1.33,2.92) | 0.175 |
| Male | Reference | 1.14 (0.91,1.43) | 1.53 (1.19,1.97) |  |
| Smoking status |  |  |  |  |
| Never | Reference | 1.42 (1.06,1.91) | 2.04 (1.47,2.83) | 0.175 |
| Previous | Reference | 1.07 (0.81,1.43) | 1.31 (0.95,1.81) |  |
| Current | Reference | 1.33 (0.76,2.35) | 1.58 (0.88,2.82) |  |
| **Bladder (C67)** |  |  |  |  |
| Age |  |  |  |  |
| ≤ 65 years | Reference | 1.46 (1.02,2.07) | 1.39 (0.93,2.08) | 0.133 |
| > 65 years | Reference | 0.92 (0.72,1.18) | 1.12 (0.84,1.49) |  |
| Sex |  |  |  |  |
| Female | Reference | 1.08 (0.68,1.71) | 1.19 (0.71,1.99) | 0.558 |
| Male | Reference | 1.00 (0.80,1.25) | 1.07 (0.82,1.39) |  |
| Smoking status |  |  |  |  |
| Never | Reference | 0.90 (0.63,1.28) | 0.95 (0.61,1.48) | 0.277 |
| Previous | Reference | 0.97 (0.73,1.29) | 1.20 (0.87,1.65) |  |
| Current | Reference | 1.41 (0.85,2.35) | 1.19 (0.68,2.07) |  |
| **CNS (C70-72)** |  |  |  |  |
| Age |  |  |  |  |
| ≤ 65 years | Reference | 1.07 (0.65,1.79) | 1.43 (0.81,2.52) | 0.760 |
| > 65 years | Reference | 1.18 (0.92,1.52) | 1.25 (0.92,1.71) |  |
| Sex |  |  |  |  |
| Female | Reference | 0.90 (0.63,1.29) | 1.00 (0.64,1.54) | 0.324 |
| Male | Reference | 1.27 (0.95,1.70) | 1.41 (1.00,2.00) |  |
| Smoking status |  |  |  |  |
| Never | Reference | 0.95 (0.69,1.31) | 1.14 (0.76,1.70) | 0.307 |
| Previous | Reference | 1.32 (0.91,1.90) | 1.49 (0.97,2.29) |  |
| Current | Reference | 1.22 (0.59,2.54) | 0.97 (0.43,2.19) |  |
| **Thyroid (C73)** |  |  |  |  |
| Age |  |  |  |  |
| ≤ 65 years | Reference | 0.86 (0.36,2.08) | 0.35 (0.10,1.25) | 0.387 |
| > 65 years | Reference | 1.14 (0.82,1.59) | 1.07 (0.71,1.62) |  |
| Sex |  |  |  |  |
| Female | Reference | 1.09 (0.75,1.57) | 0.79 (0.49,1.26) | 0.532 |
| Male | Reference | 1.07 (0.59,1.94) | 1.30 (0.64,2.65) |  |
| Smoking status |  |  |  |  |
| Never | Reference | 1.22 (0.81,1.84) | 0.99 (0.58,1.69) | 0.194 |
| Previous | Reference | 0.67 (0.39,1.15) | 0.59 (0.30,1.18) |  |
| Current | Reference | 4.17 (1.13,15.33) | 3.76 (0.92,15.43) |  |
| **Non-Hodgkin lymphoma (C82-85,96)** | |  |  |  |
| Age |  |  |  |  |
| ≤ 65 years | Reference | 1.13 (0.83,1.54) | 2.03 (1.47,2.79) | 0.059 |
| > 65 years | Reference | 1.23 (1.04,1.45) | 1.48 (1.22,1.81) |  |
| Sex |  |  |  |  |
| Female | Reference | 1.06 (0.85,1.32) | 1.36 (1.06,1.75) | 0.493 |
| Male | Reference | 1.20 (0.99,1.46) | 1.69 (1.35,2.10) |  |
| Smoking status |  |  |  |  |
| Never | Reference | 1.12 (0.92,1.37) | 1.40 (1.10,1.78) | 0.835 |
| Previous | Reference | 1.18 (0.92,1.50) | 1.67 (1.27,2.18) |  |
| Current | Reference | 1.18 (0.71,1.96) | 1.64 (0.98,2.74) |  |
| **Multiple myeloma (C90)** |  |  |  |  |
| Age |  |  |  |  |
| ≤ 65 years | Reference | 1.44 (0.91,2.28) | 0.83 (0.46,1.50) | 0.012 |
| > 65 years | Reference | 0.70 (0.54,0.93) | 0.78 (0.56,1.10) |  |
| Sex |  |  |  |  |
| Female | Reference | 0.69 (0.49,0.98) | 0.49 (0.31,0.77) | 0.386 |
| Male | Reference | 0.92 (0.68,1.25) | 0.98 (0.67,1.43) |  |
| Smoking status |  |  |  |  |
| Never | Reference | 0.67 (0.48,0.93) | 0.68 (0.45,1.04) | 0.260 |
| Previous | Reference | 0.90 (0.62,1.30) | 0.81 (0.52,1.28) |  |
| Current | Reference | 1.48 (0.66,3.34) | 0.95 (0.37,2.47) |  |
| **CLL (C91)** |  |  |  |  |
| Age |  |  |  |  |
| ≤ 65 years | Reference | 0.68 (0.42,1.10) | 0.85 (0.50,1.47) | 0.514 |
| > 65 years | Reference | 0.82 (0.63,1.07) | 0.69 (0.49,0.98) |  |
| Sex |  |  |  |  |
| Female | Reference | 0.70 (0.47,1.03) | 0.77 (0.49,1.22) | 0.525 |
| Male | Reference | 0.76 (0.57,1.02) | 0.61 (0.41,0.90) |  |
| Smoking status |  |  |  |  |
| Never | Reference | 0.66 (0.46,0.93) | 0.81 (0.53,1.23) | 0.555 |
| Previous | Reference | 0.79 (0.55,1.12) | 0.59 (0.37,0.94) |  |
| Current | Reference | 0.77 (0.35,1.67) | 0.54 (0.21,1.35) |  |

Except for subgroup variable, all analyses were adjusted for age, sex (female, male), ethnic (White, Asian, African, mixed background, unknown), education (no degree, degree, unknown), Townsend deprivation index, standing height, BMI, smoking status (never, previous, current, unknown), alcohol use (never, previous, current, unknown), physical activity (<600 MET/week, 600-3,000 MET/week, ≥3,000 MET/week), family cancer (no, yes), and assessment centre. Additionally adjusted for menopausal (no, yes, not sure, unknown), oral contraceptive use (never, ever, unknown), hormone replacement therapy (never, ever, unknown) for female. CNS, central nervous system; CLL, chronic lymphocytic leukemia.

**Table S13.** Hazard ratios for cancer outcomes among three CRP groups based on observation data by additionally adjusting for cardiovascular disease and diabetes

|  | **Low CRP (<1 mg/L)** |  | **Average CRP (1 to 3 mg/L)** | | |  | **High CRP (>3 mg/L)** | | | ***P* _trend_** |
| --- | --- | --- | --- | --- | --- | --- | --- | --- | --- | --- |
|  | **No**  **(incident cases)** |  | **No**  **(incident cases)** | **HR (95%CI)** | ***P*** |  | **No**  **(incident cases)** | **HR (95%CI)** | ***P*** |
| **Overall cancer** | 166,888 (12,380) |  | 160,143 (13,793) | 1.04 (1.02,1.07) | 0.001 |  | 93,933 (8,806) | 1.15 (1.11,1.18) | <0.001 | <0.001 |
| **Head & neck (C00-14)** | 154,674 (166) |  | 146,535 (185) | 1.06 (0.85,1.31) | 0.620 |  | 85,283 (156) | 1.46 (1.15,1.85) | 0.002 | 0.003 |
| **Esophagus (C15)** | 154,643 (136) |  | 146,558 (208) | 1.19 (0.95,1.48) | 0.126 |  | 85,273 (146) | 1.29 (1.00,1.66) | 0.046 | 0.046 |
| **Stomach (C16)** | 154,605 (97) |  | 146,482 (132) | 1.10 (0.84,1.44) | 0.501 |  | 85,232 (106) | 1.38 (1.02,1.87) | 0.034 | 0.035 |
| **Colorectal (C18-20)** | 155,467 (959) |  | 147,536 (1,186) | 1.11 (1.02,1.21) | 0.019 |  | 85,917 (791) | 1.27 (1.14,1.40) | <0.001 | <0.001 |
| **Liver (C22)** | 154,584 (76) |  | 146,453 (103) | 1.10 (0.81,1.49) | 0.546 |  | 85,230 (103) | 1.71 (1.24,2.36) | 0.001 | 0.001 |
| **Gallbladder (C23-24)** | 154,553 (45) |  | 146,399 (49) | 0.82 (0.54,1.25) | 0.364 |  | 85,172 (45) | 1.08 (0.68,1.71) | 0.747 | 0.762 |
| **Pancreas (C25)** | 154,678 (170) |  | 146,590 (240) | 1.15 (0.94,1.41) | 0.173 |  | 85,286 (159) | 1.18 (0.93,1.49) | 0.179 | 0.170 |
| **Lung (C33-34)** | 154,927 (419) |  | 147,068 (718) | 1.36 (1.20,1.54) | <0.001 |  | 85,882 (755) | 2.05 (1.80,2.33) | <0.001 | <0.001 |
| **Melanoma (C43)** | 155,064 (559) |  | 146,902 (552) | 1.01 (0.89,1.14) | 0.918 |  | 85,437 (310) | 1.04 (0.89,1.21) | 0.637 | 0.659 |
| **Non-melanotic skin (C44)** | 158,800 (4,294) |  | 150,650 (4,303) | 0.99 (0.95,1.04) | 0.725 |  | 87,430 (2,305) | 0.99 (0.94,1.05) | 0.785 | 0.755 |
| **Breast (C50)** | 83,335 (1,704) |  | 77,468 (1,940) | 1.15 (1.08,1.24) | <0.001 |  | 51,446 (1,371) | 1.21 (1.11,1.31) | <0.001 | <0.001 |
| **Uterus (C54-55)** | 81,841 (210) |  | 75,810 (281) | 1.01 (0.84,1.21) | 0.955 |  | 50,382 (306) | 1.20 (0.97,1.47) | 0.088 | 0.076 |
| **Ovary (C56)** | 81,804 (173) |  | 75,744 (215) | 1.26 (1.02,1.56) | 0.029 |  | 50,223 (147) | 1.36 (1.06,1.75) | 0.015 | 0.013 |
| **Prostate (C61)** | 74,992 (2,116) |  | 73,042 (2,221) | 0.99 (0.93,1.06) | 0.825 |  | 36,092 (1,043) | 0.94 (0.87,1.02) | 0.129 | 0.165 |
| **Kidney (C64)** | 154,689 (181) |  | 146,631 (282) | 1.26 (1.04,1.52) | 0.020 |  | 85,362 (235) | 1.63 (1.32,2.02) | <0.001 | <0.001 |
| **Bladder (C67)** | 154,689 (181) |  | 146,581 (231) | 1.01 (0.82,1.23) | 0.947 |  | 85,281 (154) | 1.09 (0.86,1.37) | 0.473 | 0.487 |
| **CNS (C70-72)** | 154,662 (154) |  | 146,523 (173) | 1.12 (0.89,1.40) | 0.341 |  | 85,232 (105) | 1.24 (0.94,1.62) | 0.124 | 0.121 |
| **Thyroid (C73)** | 154,590 (82) |  | 146,441 (91) | 1.08 (0.79,1.48) | 0.623 |  | 85,179 (52) | 0.92 (0.62,1.36) | 0.667 | 0.742 |
| **Non-Hodgkin lymphoma (C82-85,96)** | 154,862 (354) |  | 146,772 (422) | 1.14 (0.98,1.32) | 0.079 |  | 85,447 (320) | 1.53 (1.30,1.81) | <0.001 | <0.001 |
| **Multiple myeloma (C90)** | 154,670 (163) |  | 146,502 (152) | 0.82 (0.65,1.03) | 0.081 |  | 85,211 (84) | 0.74 (0.55,0.98) | 0.038 | 0.029 |
| **CLL (C91)** | 154,675 (167) |  | 146,491 (141) | 0.74 (0.59,0.93) | 0.011 |  | 85,205 (78) | 0.69 (0.51,0.92) | 0.013 | 0.007 |

Adjusted for age, sex (female, male), ethnic (White, Asian, African, mixed background, unknown), education (no degree, degree, unknown), Townsend deprivation index, standing height, BMI, smoking status (never, previous, current, unknown), alcohol use (never, previous, current, unknown), physical activity (<600 MET/week, 600-3,000 MET/week, ≥3,000 MET/week), family cancer (no, yes), assessment centre, cardiovascular, and diabetes diseases. Additionally adjusted for menopausal (no, yes, not sure, unknown), oral contraceptive use (never, ever, unknown), hormone replacement therapy (never, ever, unknown) for female. CNS, central nervous system; CLL, chronic lymphocytic leukemia.

**Table S14. Hazard ratios for cancer outcomes among three CRP groups based on observation data by additionally** **adjusting for drugs**

|  | **Low CRP (<1 mg/L)** |  | **Average CRP (1 to 3 mg/L)** | | |  | **High CRP (>3 mg/L)** | | | ***P* _trend_** |
| --- | --- | --- | --- | --- | --- | --- | --- | --- | --- | --- |
|  | **No**  **(incident cases)** |  | **No**  **(incident cases)** | **HR (95%CI)** | ***P*** |  | **No**  **(incident cases)** | **HR (95%CI)** | ***P*** |
| **Overall cancer** | 166,888 (12,380) |  | 160,143 (13,793) | 1.05 (1.02,1.07) | <0.001 |  | 93,933 (8,806) | 1.15 (1.12,1.19) | <0.001 | <0.001 |
| **Head & neck (C00-14)** | 154,674 (166) |  | 146,535 (185) | 1.06 (0.85,1.31) | 0.627 |  | 85,283 (156) | 1.46 (1.15,1.85) | 0.002 | 0.003 |
| **Esophagus (C15)** | 154,643 (136) |  | 146,558 (208) | 1.19 (0.95,1.49) | 0.121 |  | 85,273 (146) | 1.30 (1.01,1.67) | 0.042 | 0.042 |
| **Stomach (C16)** | 154,605 (97) |  | 146,482 (132) | 1.11 (0.85,1.45) | 0.457 |  | 85,232 (106) | 1.40 (1.04,1.89) | 0.027 | 0.028 |
| **Colorectal (C18-20)** | 155,467 (959) |  | 147,536 (1,186) | 1.11 (1.02,1.21) | 0.019 |  | 85,917 (791) | 1.27 (1.14,1.40) | <0.001 | <0.001 |
| **Liver (C22)** | 154,584 (76) |  | 146,453 (103) | 1.11 (0.82,1.50) | 0.513 |  | 85,230 (103) | 1.75 (1.26,2.41) | 0.001 | 0.001 |
| **Gallbladder (C23-24)** | 154,553 (45) |  | 146,399 (49) | 0.82 (0.54,1.24) | 0.343 |  | 85,172 (45) | 1.06 (0.67,1.67) | 0.815 | 0.828 |
| **Pancreas (C25)** | 154,678 (170) |  | 146,590 (240) | 1.16 (0.94,1.42) | 0.163 |  | 85,286 (159) | 1.19 (0.93,1.50) | 0.160 | 0.152 |
| **Lung (C33-34)** | 154,927 (419) |  | 147,068 (718) | 1.38 (1.22,1.56) | <0.001 |  | 85,882 (755) | 2.10 (1.84,2.38) | <0.001 | <0.001 |
| **Melanoma (C43)** | 155,064 (559) |  | 146,902 (552) | 1.01 (0.89,1.14) | 0.926 |  | 85,437 (310) | 1.04 (0.89,1.21) | 0.649 | 0.671 |
| **Non-melanotic skin (C44)** | 158,800 (4,294) |  | 150,650 (4,303) | 0.99 (0.95,1.04) | 0.767 |  | 87,430 (2,305) | 0.99 (0.94,1.05) | 0.840 | 0.812 |
| **Breast (C50)** | 83,335 (1,704) |  | 77,468 (1,940) | 1.15 (1.08,1.23) | <0.001 |  | 51,446 (1,371) | 1.20 (1.11,1.31) | <0.001 | <0.001 |
| **Uterus (C54,55)** | 81,841 (210) |  | 75,810 (281) | 1.01 (0.84,1.21) | 0.938 |  | 50,382 (306) | 1.20 (0.98,1.47) | 0.079 | 0.068 |
| **Ovary (C56)** | 81,804 (173) |  | 75,744 (215) | 1.27 (1.03,1.56) | 0.028 |  | 50,223 (147) | 1.37 (1.07,1.76) | 0.014 | 0.011 |
| **Prostate (C61)** | 74,992 (2,116) |  | 73,042 (2,221) | 0.99 (0.93,1.06) | 0.825 |  | 36,092 (1,043) | 0.94 (0.87,1.02) | 0.129 | 0.165 |
| **Kidney (C64)** | 154,689 (181) |  | 146,631 (282) | 1.27 (1.05,1.54) | 0.014 |  | 85,362 (235) | 1.66 (1.35,2.06) | <0.001 | <0.001 |
| **Bladder (C67)** | 154,689 (181) |  | 146,581 (231) | 1.01 (0.83,1.23) | 0.917 |  | 85,281 (154) | 1.10 (0.87,1.38) | 0.431 | 0.444 |
| **CNS (C70-72)** | 154,662 (154) |  | 146,523 (173) | 1.11 (0.88,1.39) | 0.378 |  | 85,232 (105) | 1.22 (0.93,1.60) | 0.149 | 0.147 |
| **Thyroid (C73)** | 154,590 (82) |  | 146,441 (91) | 1.09 (0.80,1.49) | 0.586 |  | 85,179 (52) | 0.93 (0.63,1.38) | 0.727 | 0.806 |
| **Non-Hodgkin lymphoma (C82-85,96)** | 154,862 (354) |  | 146,772 (422) | 1.14 (0.99,1.32) | 0.071 |  | 85,447 (320) | 1.54 (1.31,1.82) | <0.001 | <0.001 |
| **Multiple myeloma (C90)** | 154,670 (163) |  | 146,502 (152) | 0.81 (0.65,1.03) | 0.081 |  | 85,211 (84) | 0.74 (0.55,0.98) | 0.038 | 0.029 |
| **CLL (C91)** | 154,675 (167) |  | 146,491 (141) | 0.73 (0.58,0.93) | 0.009 |  | 85,205 (78) | 0.68 (0.51,0.91) | 0.010 | 0.005 |

Adjusted for age, sex (female, male), ethnic (White, Asian, African, mixed background, unknown), education (no degree, degree, unknown), Townsend deprivation index, standing height, BMI, smoking status (never, previous, current, unknown), alcohol use (never, previous, current, unknown), physical activity (<600 MET/week, 600-3,000 MET/week, ≥3,000 MET/week), family cancer (no, yes), assessment centre, cardiovascular, diabetes diseases, regular use of aspirin and ibuprofen. Additionally adjusted for menopausal (no, yes, not sure, unknown), oral contraceptive use (never, ever, unknown), hormone replacement therapy (never, ever, unknown) for female. CNS, central nervous system; CLL, chronic lymphocytic leukemia.

**Table S15.** Hazard ratios for cancer outcomes among three CRP groups based on observation data by excluding of patients diagnosed in the first two follow-up

|  | **Low CRP (<1 mg/L)** |  | **Average CRP (1 to 3 mg/L)** | | |  | **High CRP (>3 mg/L)** | | | ***P* _trend_** |
| --- | --- | --- | --- | --- | --- | --- | --- | --- | --- | --- |
|  | **No**  **(incident cases)** |  | **No**  **(incident cases)** | **HR (95%CI)** | ***P*** |  | **No**  **(incident cases)** | **HR (95%CI)** | ***P*** |
| **Overall cancer** | 164,085 (9,577) |  | 156,864 (10,514) | 1.02 (1.00,1.05) | 0.105 |  | 91,579 (6,452) | 1.08 (1.04,1.12) | <0.001 | <0.001 |
| **Head & neck (C00-14)** | 154,637 (129) |  | 146,493 (143) | 1.04 (0.81,1.32) | 0.780 |  | 85,249 (122) | 1.43 (1.09,1.87) | 0.010 | 0.012 |
| **Esophagus (C15)** | 154,618 (111) |  | 146,516 (166) | 1.14 (0.89,1.46) | 0.293 |  | 85,235 (108) | 1.13 (0.85,1.51) | 0.398 | 0.389 |
| **Stomach (C16)** | 154,593 (85) |  | 146,450 (100) | 0.93 (0.69,1.25) | 0.608 |  | 85,200 (74) | 1.05 (0.75,1.48) | 0.769 | 0.799 |
| **Colorectal (C18-20)** | 155,270 (762) |  | 147,275 (925) | 1.07 (0.97,1.18) | 0.173 |  | 85,669 (543) | 1.05 (0.93,1.19) | 0.404 | 0.347 |
| **Liver (C22)** | 154,570 (62) |  | 146,438 (88) | 1.11 (0.80,1.55) | 0.535 |  | 85,204 (77) | 1.49 (1.03,2.15) | 0.033 | 0.032 |
| **Gallbladder (C23-24)** | 154,549 (41) |  | 146,389 (39) | 0.69 (0.44,1.09) | 0.113 |  | 85,160 (33) | 0.81 (0.48,1.35) | 0.412 | 0.378 |
| **Pancreas (C25)** | 154,651 (143) |  | 146,549 (199) | 1.12 (0.90,1.40) | 0.303 |  | 85,246 (119) | 1.02 (0.78,1.34) | 0.872 | 0.822 |
| **Lung (C33-34)** | 154,856 (348) |  | 146,939 (589) | 1.34 (1.17,1.53) | <0.001 |  | 85,704 (577) | 1.88 (1.63,2.17) | <0.001 | <0.001 |
| **Melanoma (C43)** | 154,946 (441) |  | 146,744 (394) | 0.90 (0.78,1.04) | 0.142 |  | 85,366 (239) | 1.00 (0.84,1.19) | 0.996 | 0.783 |
| **Non-melanotic skin (C44)** | 157,861 (3,355) |  | 149,719 (3,372) | 0.99 (0.94,1.04) | 0.746 |  | 86,938 (1,813) | 1.00 (0.94,1.06) | 0.897 | 0.859 |
| **Breast (C50)** | 82,905 (1,274) |  | 76,985 (1,457) | 1.17 (1.08,1.26) | <0.001 |  | 51,075 (1,000) | 1.18 (1.07,1.30) | 0.001 | <0.001 |
| **Uterus (C54-55)** | 81,795 (164) |  | 75,737 (208) | 0.94 (0.76,1.17) | 0.593 |  | 50,302 (226) | 1.10 (0.87,1.40) | 0.417 | 0.382 |
| **Ovary (C56)** | 81,760 (129) |  | 75,694 (165) | 1.29 (1.01,1.64) | 0.038 |  | 50,176 (100) | 1.23 (0.92,1.65) | 0.169 | 0.130 |
| **Prostate (C61)** | 74,519 (1,643) |  | 72,520 (1,699) | 0.98 (0.92,1.06) | 0.651 |  | 35,815 (766) | 0.90 (0.82,0.98) | 0.019 | 0.031 |
| **Kidney (C64)** | 154,654 (146) |  | 146,588 (239) | 1.30 (1.05,1.61) | 0.014 |  | 85,296 (169) | 1.41 (1.11,1.80) | 0.005 | 0.005 |
| **Bladder (C67)** | 154,638 (130) |  | 146,528 (178) | 1.07 (0.85,1.35) | 0.588 |  | 85,239 (112) | 1.07 (0.82,1.41) | 0.611 | 0.601 |
| **CNS (C70-72)** | 154,631 (123) |  | 146,477 (127) | 1.01 (0.78,1.30) | 0.960 |  | 85,209 (82) | 1.18 (0.87,1.60) | 0.288 | 0.326 |
| **Thyroid (C73)** | 154,571 (63) |  | 146,413 (63) | 0.96 (0.67,1.39) | 0.837 |  | 85,167 (40) | 0.90 (0.57,1.40) | 0.630 | 0.635 |
| **Non-Hodgkin lymphoma (C82-85,96)** | 154,798 (290) |  | 146,685 (335) | 1.09 (0.93,1.29) | 0.283 |  | 85,343 (216) | 1.24 (1.02,1.51) | 0.027 | 0.029 |
| **Multiple myeloma (C90)** | 154,646 (139) |  | 146,470 (120) | 0.75 (0.58,0.97) | 0.029 |  | 85,194 (67) | 0.68 (0.50,0.94) | 0.020 | 0.012 |
| **CLL (C91)** | 154,636 (128) |  | 146,448 (98) | 0.68 (0.52,0.89) | 0.005 |  | 85,189 (62) | 0.73 (0.52,1.01) | 0.060 | 0.027 |

Adjusted for age, sex (female, male), ethnic (White, Asian, African, mixed background, unknown), education (no degree, degree, unknown), Townsend deprivation index, standing height, BMI, smoking status (never, previous, current, unknown), alcohol use (never, previous, current, unknown), physical activity (<600 MET/week, 600-3,000 MET/week, ≥3,000 MET/week), family cancer (no, yes), and assessment centre. Additionally adjusted for menopausal (no, yes, not sure, unknown), oral contraceptive use (never, ever, unknown), hormone replacement therapy (never, ever, unknown) for female. CNS, central nervous system; CLL, chronic lymphocytic leukemia.

**Table S16. Hazard ratios for cancer outcomes among three CRP groups based on observation data by only including of patients diagnosed in the first two follow-up**

|  | **Low CRP (<1 mg/L)** |  | **Average CRP (1 to 3 mg/L)** | | |  | **High CRP (>3 mg/L)** | | | ***P* _trend_** |
| --- | --- | --- | --- | --- | --- | --- | --- | --- | --- | --- |
|  | **No (incident cases)** |  | **No (incident cases)** | **HR (95%CI)** | ***P*** |  | **No (incident cases)** | **HR (95%CI)** | ***P*** |
| **Overall cancer** | 157,311 (2,803) |  | 149,629 (3,279) | 1.12 (1.06,1.18) | <0.001 |  | 87,481 (2,354) | 1.41 (1.33,1.49) | <0.001 | <0.001 |
| **Head & neck (C00-14)** | 154,545 (37) |  | 146,392 (42) | 1.14 (0.72,1.80) | 0.584 |  | 85,161 (34) | 1.57 (0.95,2.61) | 0.081 | 0.086 |
| **Esophagus (C15)** | 154,532 (25) |  | 146,392 (42) | 1.43 (0.86,2.37) | 0.167 |  | 85,165 (38) | 2.16 (1.26,3.70) | 0.005 | 0.005 |
| **Stomach (C16)** | 154,520 (12) |  | 146,382 (32) | 2.36 (1.20,4.63) | 0.013 |  | 85,158 (32) | 3.90 (1.93,7.89) | <0.001 | <0.001 |
| **Colorectal (C18-20)** | 154,705 (197) |  | 146,611 (261) | 1.25 (1.04,1.52) | 0.020 |  | 85,374 (248) | 2.16 (1.76,2.64) | <0.001 | <0.001 |
| **Liver (C22)** | 154,522 (14) |  | 146,365 (15) | 1.04 (0.49,2.19) | 0.923 |  | 85,153 (26) | 3.10 (1.53,6.29) | 0.002 | 0.001 |
| **Gallbladder (C23-24)** | 154,512 (4) |  | 146,360 (10) | 2.12 (0.65,6.90) | 0.212 |  | 85,139 (12) | 3.98 (1.18,13.38) | 0.026 | 0.020 |
| **Pancreas (C25)** | 154,535 (27) |  | 146,391 (41) | 1.30 (0.79,2.14) | 0.300 |  | 85,167 (40) | 2.08 (1.22,3.54) | 0.007 | 0.006 |
| **Lung (C33-34)** | 154,579 (71) |  | 146,479 (129) | 1.53 (1.13,2.05) | 0.005 |  | 85,305 (178) | 3.13 (2.33,4.20) | <0.001 | <0.001 |
| **Melanoma (C43)** | 154,623 (118) |  | 146,508 (158) | 1.41 (1.10,1.81) | 0.007 |  | 85,198 (71) | 1.16 (0.84,1.61) | 0.355 | 0.188 |
| **Non-melanotic skin (C44)** | 155,445 (939) |  | 147,278 (931) | 1.00 (0.91,1.09) | 0.925 |  | 85,617 (492) | 0.98 (0.87,1.11) | 0.785 | 0.793 |
| **Breast (C50)** | 82,061 (430) |  | 76,011 (483) | 1.12 (0.98,1.28) | 0.103 |  | 50,446 (371) | 1.28 (1.09,1.50) | 0.003 | 0.003 |
| **Uterus (C54-55)** | 81,677 (46) |  | 75,602 (73) | 1.22 (0.84,1.79) | 0.299 |  | 50,156 (80) | 1.53 (1.01,2.33) | 0.044 | 0.042 |
| **Ovary (C56)** | 81,675 (44) |  | 75,579 (50) | 1.19 (0.78,1.81) | 0.429 |  | 50,123 (47) | 1.78 (1.12,2.85) | 0.015 | 0.016 |
| **Prostate (C61)** | 73,349 (473) |  | 71,343 (522) | 1.02 (0.90,1.16) | 0.718 |  | 35,326 (277) | 1.08 (0.93,1.27) | 0.31 | 0.327 |
| **Kidney (C64)** | 154,543 (35) |  | 146,392 (43) | 1.05 (0.67,1.66) | 0.836 |  | 85,193 (66) | 2.68 (1.71,4.19) | <0.001 | <0.001 |
| **Bladder (C67)** | 154,559 (51) |  | 146,403 (53) | 0.86 (0.58,1.28) | 0.458 |  | 85,169 (42) | 1.16 (0.75,1.80) | 0.506 | 0.564 |
| **CNS (C70-72)** | 154,539 (31) |  | 146,396 (46) | 1.56 (0.97,2.50) | 0.066 |  | 85,150 (23) | 1.44 (0.81,2.58) | 0.219 | 0.165 |
| **Thyroid (C73)** | 154,527 (19) |  | 146,378 (28) | 1.52 (0.82,2.79) | 0.181 |  | 85,139 (12) | 1.02 (0.46,2.29) | 0.957 | 0.796 |
| **Non-Hodgkin lymphoma**  **(C82-85,96)** | 154,572 (64) |  | 146,437 (87) | 1.34 (0.96,1.87) | 0.081 |  | 85,231 (104) | 2.90 (2.07,4.07) | <0.001 | <0.001 |
| **Multiple myeloma (C90)** | 154,531 (24) |  | 146,382 (32) | 1.17 (0.68,2.02) | 0.580 |  | 85,144 (17) | 1.05 (0.53,2.07) | 0.891 | 0.844 |
| **CLL (C91)** | 154,547 (39) |  | 146,393 (43) | 0.93 (0.59,1.45) | 0.740 |  | 85,143 (16) | 0.56 (0.30,1.05) | 0.070 | 0.092 |

Adjusted for age, sex (female, male), ethnic (White, Asian, African, mixed background, unknown), education (no degree, degree, unknown), Townsend deprivation index, standing height, BMI, smoking status (never, previous, current, unknown), alcohol use (never, previous, current, unknown), physical activity (<600 MET/week, 600-3,000 MET/week, ≥3,000 MET/week), family cancer (no, yes), and assessment centre. Additionally adjusted for menopausal (no, yes, not sure, unknown), oral contraceptive use (never, ever, unknown), hormone replacement therapy (never, ever, unknown) for female. CNS, central nervous system; CLL, chronic lymphocytic leukemia.

**Table S17.** Hazard ratios for cancer outcomes among three CRP groups based on observation data by excluding of participants with CRP > 10 mg/L

|  | **Low CRP (<1 mg/L)** |  | **Average CRP (1 to 3 mg/L)** | | |  | **High CRP (>3 mg/L)** | | | ***P* _trend_** |
| --- | --- | --- | --- | --- | --- | --- | --- | --- | --- | --- |
|  | **No**  **(incident cases)** |  | **No**  **(incident cases)** | **HR (95%CI)** | ***P*** |  | **No**  **(incident cases)** | **HR (95%CI)** | ***P*** |
| **Overall cancer** | 166,888 (12,380) |  | 160,143 (13,793) | 1.05 (1.02,1.07) | <0.001 |  | 77,036 (7,100) | 1.13 (1.09,1.17) | <0.001 | <0.001 |
| **Head & neck (C00-14)** | 154,674 (166) |  | 146,535 (185) | 1.05 (0.84,1.30) | 0.671 |  | 70,056 (120) | 1.36 (1.05,1.76) | 0.019 | 0.026 |
| **Esophagus (C15)** | 154,643 (136) |  | 146,558 (208) | 1.17 (0.94,1.46) | 0.169 |  | 70,049 (113) | 1.19 (0.91,1.56) | 0.194 | 0.176 |
| **Stomach (C16)** | 154,605 (97) |  | 146,482 (132) | 1.10 (0.84,1.44) | 0.509 |  | 70,022 (86) | 1.36 (0.99,1.87) | 0.055 | 0.060 |
| **Colorectal (C18-20)** | 155,467 (959) |  | 147,536 (1,186) | 1.12 (1.02,1.22) | 0.015 |  | 70,566 (631) | 1.24 (1.11,1.38) | <0.001 | <0.001 |
| **Liver (C22)** | 154,584 (76) |  | 146,453 (103) | 1.08 (0.79,1.46) | 0.628 |  | 70,018 (82) | 1.62 (1.15,2.28) | 0.006 | 0.006 |
| **Gallbladder (C23-24)** | 154,553 (45) |  | 146,399 (49) | 0.82 (0.54,1.24) | 0.348 |  | 69,978 (42) | 1.23 (0.77,1.95) | 0.388 | 0.431 |
| **Pancreas (C25)** | 154,678 (170) |  | 146,590 (240) | 1.15 (0.94,1.41) | 0.166 |  | 70,072 (136) | 1.24 (0.97,1.59) | 0.086 | 0.079 |
| **Lung (C33-34)** | 154,927 (419) |  | 147,068 (718) | 1.37 (1.21,1.55) | <0.001 |  | 70,506 (570) | 1.94 (1.70,2.22) | <0.001 | <0.001 |
| **Melanoma (C43)** | 155,064 (559) |  | 146,902 (552) | 1.01 (0.89,1.14) | 0.936 |  | 70,185 (249) | 1.00 (0.85,1.18) | 0.954 | 0.945 |
| **Non-melanotic skin (C44)** | 158,800 (4,294) |  | 150,650 (4,303) | 0.99 (0.95,1.04) | 0.697 |  | 71,800 (1,866) | 0.97 (0.92,1.03) | 0.365 | 0.381 |
| **Breast (C50)** | 83,335 (1,704) |  | 77,468 (1,940) | 1.14 (1.07,1.23) | <0.001 |  | 42,183 (1,141) | 1.20 (1.11,1.31) | <0.001 | <0.001 |
| **Uterus (C54-55)** | 81,841 (210) |  | 75,810 (281) | 1.01 (0.84,1.22) | 0.897 |  | 41,285 (242) | 1.21 (0.98,1.50) | 0.078 | 0.076 |
| **Ovary (C56)** | 81,804 (173) |  | 75,744 (215) | 1.28 (1.04,1.58) | 0.022 |  | 41,164 (121) | 1.40 (1.08,1.81) | 0.012 | 0.008 |
| **Prostate (C61)** | 74,992 (2,116) |  | 73,042 (2,221) | 0.99 (0.93,1.05) | 0.796 |  | 29,752 (861) | 0.94 (0.87,1.03) | 0.180 | 0.228 |
| **Kidney (C64)** | 154,689 (181) |  | 146,631 (282) | 1.24 (1.02,1.50) | 0.028 |  | 70,113 (177) | 1.47 (1.17,1.84) | 0.001 | 0.001 |
| **Bladder (C67)** | 154,689 (181) |  | 146,581 (231) | 1.02 (0.83,1.24) | 0.875 |  | 70,060 (124) | 1.09 (0.85,1.39) | 0.485 | 0.507 |
| **CNS (C70-72)** | 154,662 (154) |  | 146,523 (173) | 1.12 (0.89,1.40) | 0.326 |  | 70,028 (92) | 1.32 (1.00,1.75) | 0.054 | 0.057 |
| **Thyroid (C73)** | 154,590 (82) |  | 146,441 (91) | 1.09 (0.80,1.50) | 0.577 |  | 69,978 (42) | 0.93 (0.62,1.41) | 0.744 | 0.861 |
| **Non-Hodgkin lymphoma**  **(C82-85,96)** | 154,862 (354) |  | 146,772 (422) | 1.13 (0.98,1.31) | 0.097 |  | 70,191 (255) | 1.46 (1.22,1.74) | <0.001 | <0.001 |
| **Multiple myeloma (C90)** | 154,670 (163) |  | 146,502 (152) | 0.81 (0.64,1.02) | 0.075 |  | 70,009 (73) | 0.77 (0.57,1.04) | 0.092 | 0.060 |
| **CLL (C91)** | 154,675 (167) |  | 146,491 (141) | 0.74 (0.59,0.94) | 0.012 |  | 70,002 (66) | 0.71 (0.52,0.97) | 0.032 | 0.013 |

Adjusted for age, sex (female, male), ethnic (White, Asian, African, mixed background, unknown), education (no degree, degree, unknown), Townsend deprivation index, standing height, BMI, smoking status (never, previous, current, unknown), alcohol use (never, previous, current, unknown), physical activity (<600 MET/week, 600-3,000 MET/week, ≥3,000 MET/week), family cancer (no, yes), and assessment centre. Additionally adjusted for menopausal (no, yes, not sure, unknown), oral contraceptive use (never, ever, unknown), hormone replacement therapy (never, ever, unknown) for female. CNS, central nervous system; CLL, chronic lymphocytic leukemia.
