# Supplementary material for: C-reactive protein and cancer risk: a pan-cancer study of prospective cohort and Mendelian randomization analysis
Source: BMC Med. 2022 Sep 19;20:301. doi: 10.1186/s12916-022-02506-x (PMC9484145; doi:10.1186/s12916-022-02506-x)
Supplement: Supplementary file 3 — Additional file 3: Figure S1. Analysis of the shape of the relationship between CRP and cancer outcomes by excluding of patients diagnosed in the first two follow-up. Figure S2. Analysis of the shape of the relationship between log-transformed CRP concentration and cancer outcomes. Figure S3. Sensitivity analysis of the linear MR analysis of log-transformed CRP and cancer risks. Figure S4. Sensitivity analysis of the linear MR analysis in people genetically confirmed of British ancestry. Figure S5. Sensitivity analysis of the linear MR analysis using rs2794520 as instrument variable. [file 12916_2022_2506_MOESM3_ESM.doc]

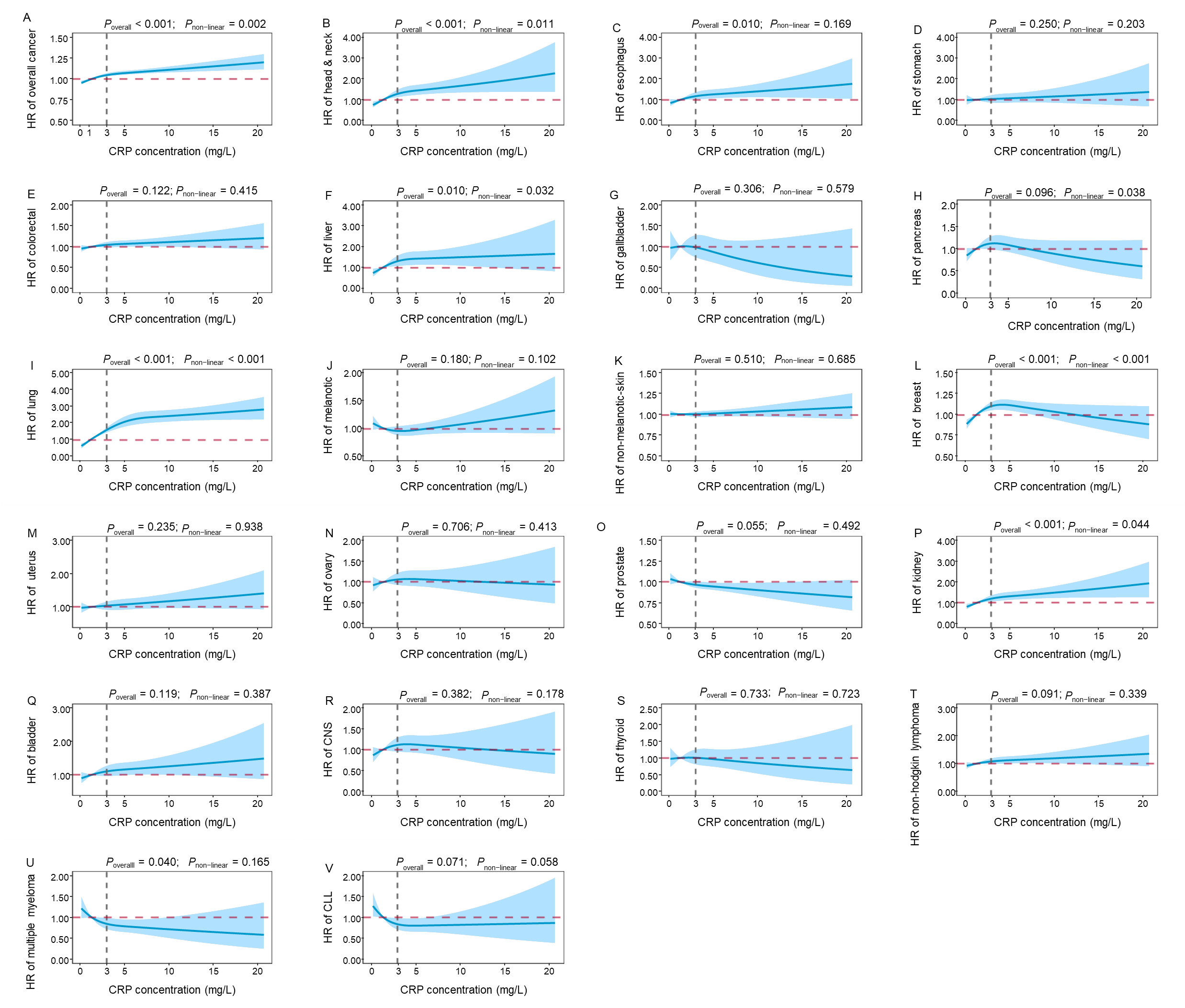
**Figure S1.** Analysis of the shape of the relationship between CRP and cancer outcomes by excluding of patients diagnosed in the first two follow-up

Adjusted for age, sex (female, male), ethnic (White, Asian, African, mixed background, unknown), education (no degree, degree, unknown), Townsend deprivation index, standing height, BMI, smoking status (never, previous, current, unknown), alcohol use (never, previous, current, unknown), physical activity (<600 MET/week, 600-3,000 MET/week, ≥3,000 MET/week), family cancer (no, yes), and assessment centre. Additionally, adjusted for menopausal (no, yes, not sure, unknown), oral contraceptive use (never, ever, unknown), hormone replacement therapy (never, ever, unknown) for female. CNS, central nervous system; CLL, chronic lymphocytic leukemia

**
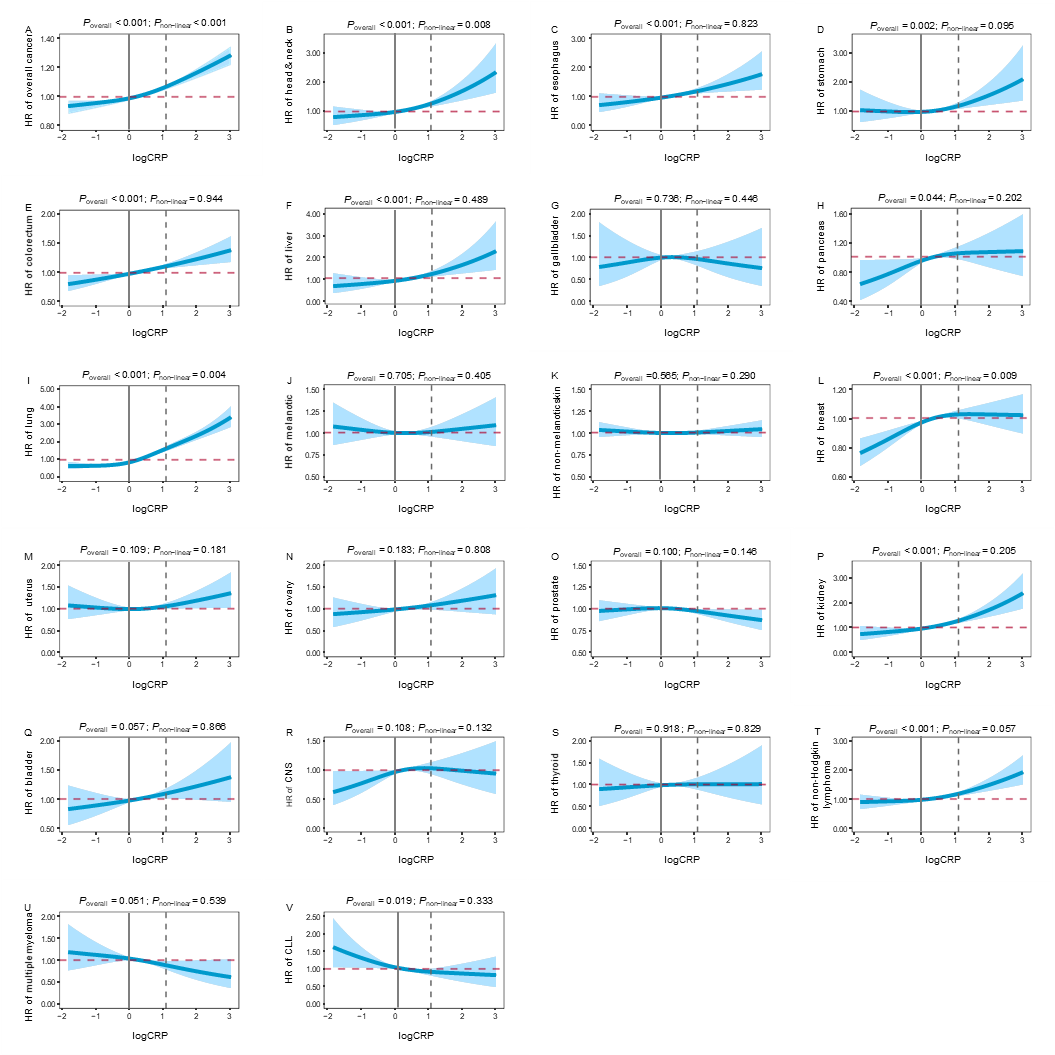
**

**Figure S2.** Analysis of the shape of the relationship between log-transformed CRP concentration and cancer outcomes

The grey solid and dashed lines represent the value of log 1 and log 3, respectively.

Adjusted for age, sex (female, male), ethnic (White, Asian, African, mixed background, unknown), education (no degree, degree, unknown), Townsend deprivation index, standing height, BMI, smoking status (never, previous, current, unknown), alcohol use (never, previous, current, unknown), physical activity (<600 MET/week, 600-3,000 MET/week, ≥3,000 MET/week), family cancer (no, yes), and assessment centre. Additionally, adjusted for menopausal (no, yes, not sure, unknown), oral contraceptive use (never, ever, unknown), hormone replacement therapy (never, ever, unknown) for female. CNS, central nervous system; CLL, chronic lymphocytic leukemia

**
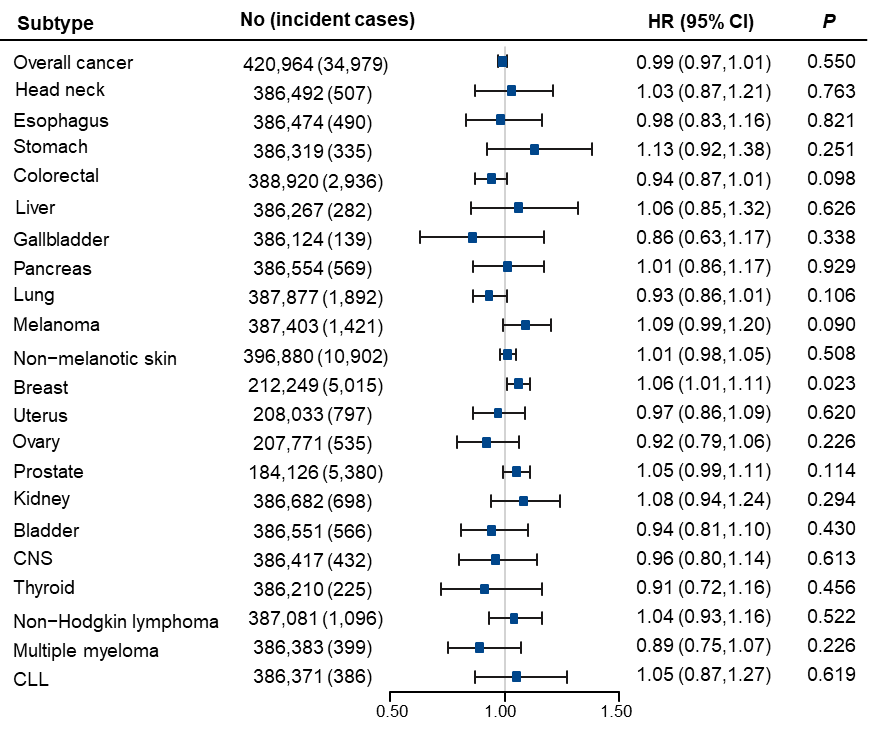
**

**Figure S3.** Sensitivity analysis of the linear MR analysis of log-transformed CRP and cancer risks

Adjusted for age, sex (female, male), BMI, smoking status (never, previous, current, unknown), top ten genetic principal components, and chip. CNS, central nervous system; CLL, chronic lymphocytic leukemia

**
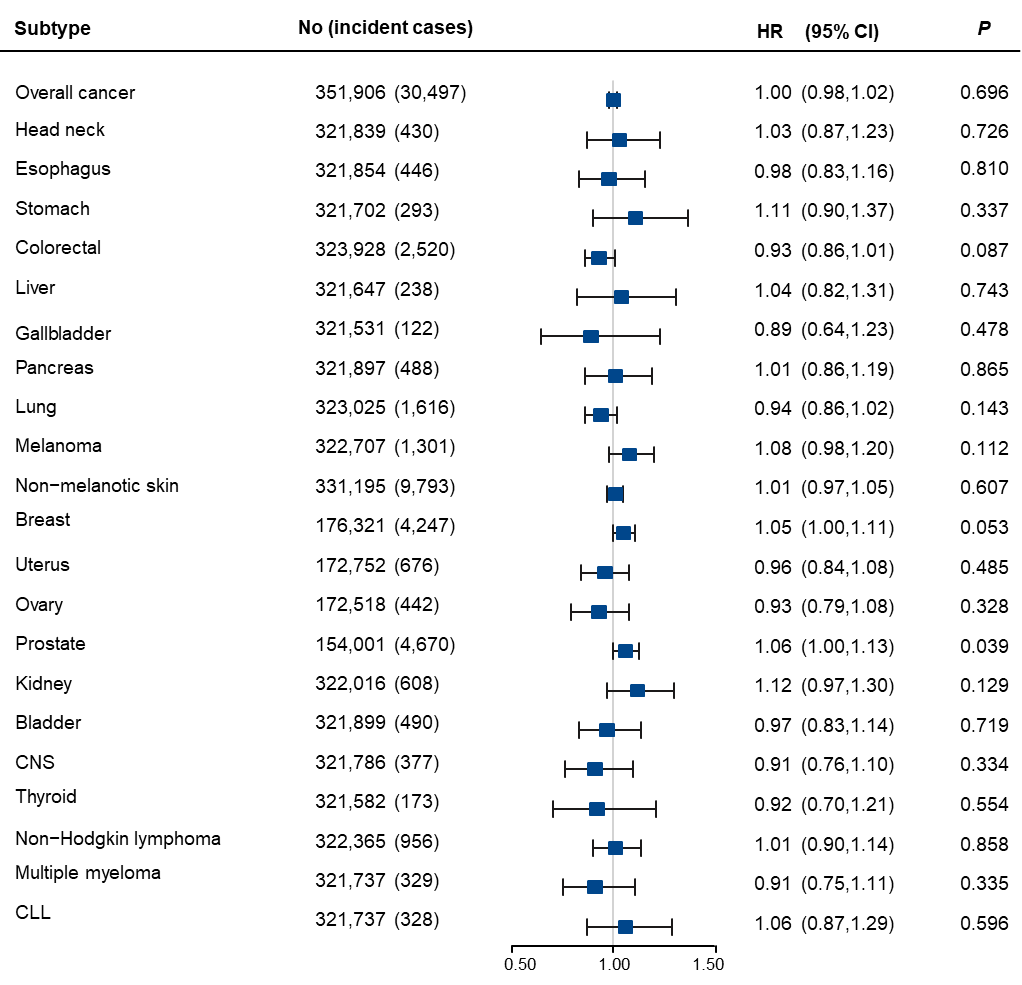
**

**Figure S4.** Sensitivity analysis of the linear MR analysis in people genetically confirmed of British ancestry

Adjusted for age, sex (female, male), BMI, smoking status (never, previous, current, unknown), top ten genetic principal components, and chip. CNS, central nervous system; CLL, chronic lymphocytic leukemia





**Figure S5.** Sensitivity analysis of the linear MR analysis using rs2794520 as instrument variable

Adjusted for age, sex (female, male), BMI, smoking status (never, previous, current, unknown), top ten genetic principal components, and chip. CNS, central nervous system; CLL, chronic lymphocytic leukemia
